# The Synthesis, In Vitro Bio-Evaluation, and In Silico Molecular Docking Studies of Pyrazoline–Thiazole Hybrid Analogues as Promising Anti-α-Glucosidase and Anti-Urease Agents

**DOI:** 10.3390/ph16121650

**Published:** 2023-11-25

**Authors:** Yousaf Khan, Shoaib Khan, Rafaqat Hussain, Aneela Maalik, Wajid Rehman, Mohamed W. Attwa, Rafia Masood, Hany W. Darwish, Hazem A. Ghabbour

**Affiliations:** 1 Department of Chemistry, COMSATS University Islamabad Campus, Islamabad 45550, Pakistan; yousaf7n@gmail.com (Y.K.); rafia12@yahoo.com (R.M.); 2 Department of Chemistry, Abbottabad University of Science and Technology (AUST), Abbottabad 22500, Pakistan; shoaibkhanswati@gmail.com; 3 Department of Chemistry, Hazara University, Mansehra 21120, Pakistan; sonowaj@yahoo.com; 4 Department of Pharmaceutical Chemistry, College of Pharmacy, King Saud University, Riyadh 11451, Saudi Arabia; mzeidan@ksu.edu.sa (M.W.A.);; 5 School of Health and Biomedical Sciences, RMIT University, Melbourne 3083, Australia; hazem.ghabbour@rmit.edu.au

**Keywords:** synthesis, pyrazoline based thiadiazole, SAR, *α*-glucosidase, urease, molecular modelling studies

## Abstract

In the present work, a concise library of benzothiazole-derived pyrazoline-based thiazole (**1**–**17**) was designed and synthesized by employing a multistep reaction strategy. The newly synthesized compounds were screened for their α-glucosidase and urease inhibitory activities. The scaffolds (**1**–**17**) were characterized using a combination of several spectroscopic techniques, including FT-IR, ^1^H-NMR, ^13^C-NMR, and EI-MS. The majority of the synthesized compounds demonstrated a notable potency against *α*-glucosidase and urease enzymes. These analogues disclosed varying degrees of *α*-glucosidase and urease inhibitory activities, with their IC_50_ values ranging from 2.50 to 17.50 μM (*α*-glucosidase) and 14.30 to 41.50 (urease). Compounds **6**, **7**, **14**, and **12**, with IC_50_ values of 2.50, 3.20, 3.40, and 3.50 μM as compared to standard acarbose (IC_50_ = 5.30 µM), while the same compounds showed 14.30, 19.20, 21.80, and 22.30 comparable with thiourea (IC_50_ = 31.40 μM), respectively, showed excellent inhibitory activity. The structure−activity relationship revealed that the size and electron-donating or electron-withdrawing effects of substituents influenced the enzymatic activities such as α-glucosidase and urease. Compound **6** was a dual potent inhibitor against *α*-glucosidase and urease due to the presence of -CF_3_ electron-withdrawing functionality on the phenyl ring. To the best of our knowledge, these synthetic compounds were found to be the most potent dual inhibitors of α-glucosidase and urease with minimum IC_50_ values. Moreover, in silico studies on most active compounds, i.e., **6**, **7**, **14**, and **12**, were also performed to understand the binding interaction of most active compounds with active sites of α-glucosidase and urease enzymes.

## 1. Introduction

The discovery of new drugs is often facilitated by the inhibition of enzymes. Urease and *α*-glucosidase play important roles in a variety of medical applications. There are vital enzymes that serve an integral role in various clinical fields [1,2]. There are millions of people worldwide affected by diabetes mellitus (DM), a metabolic disorder caused by high blood sugar levels [3]. A number of complications are associated with diabetes mellitus metabolic disease, such as cardiovascular disorders, reduced wound healing ability, and cancer, as well as severe health problems such as retinopathy, neuropathy, and amputations. Diabetes mellitus can be cured largely through the control of glycemia [4,5,6]. There is a significant amount of *α*-glucosidase present on the epithelial walls of the small intestines. Carbohydrate metabolism is regulated by it. It is also beneficial to treat diabetes by inhibiting this enzyme, since it reduces the blood’s glucose level [7,8]. Miglitol, acarbose, and voglibose are a few of the medications that can cause flatulence, diarrhoea, and gastrointestinal disease [9,10]. Additionally, it contributes to the conversion of oligosaccharides into simple sugar via hydrolysis in the salivary glands and pancreas. The treatment of diabetes is therefore enhanced by its ability to lower the postprandial blood sugar levels. The high bioavailability of pyrazoline in the organic realm has led to the development of quick, efficient, and environmentally friendly methods for synthesizing it [11].

Urease belongs to the super family of phosphodiesterase and amidohydrolase, which initiate the degradation of urea into ammonia and carbon dioxide. Eukaryotes and prokaryotes both produce urease enzymes. In response to the hyperactivity of the urease enzyme, excessive amounts of ammonia are released into the stomach, causing alkalinity and helping to improve mucosal permeability [12,13,14,15]. Even though humans lack urease enzymes, they synthesize urea as an end product of protein metabolism and eliminate it through urine [16,17,18]. Urease enzymes facilitate monitoring and regulating the nitrogen metabolism in cattle and many other animals. In addition to causing bacterial infections, high levels of these enzymes can cause various pathogenic conditions. In humans, low stomach pH eases the growth of *Helicobacter pylori* (HP). In the long run, it causes ulcers in the stomach and intestines, and ultimately may lead to cancer [19,20,21,22,23].

Individuals who concurrently have diabetes mellitus and peptic ulcers are advised to employ a variety of medical therapies. The coexistence of a peptic ulcer can potentially amplify health concerns in patients diagnosed with diabetes due to the commonly seen phenomenon of delayed wound healing. A proposed approach has been formulated to address the management of individuals who have both diabetes mellitus and peptic ulcers. This technique aims to simultaneously target the activities of α-glucosidase and urease. This technique holds significant potential as a therapeutic alternative for people with DM and peptic ulcers [24,25].

Pyrazole-based compounds have garnered significant attention owing to their diverse antineoplastic properties, including chemotherapy. The presence of a pyrazole core has been recognized as a significant contributor to the augmented cytotoxicity of chalcones [26].

In recent times, there has been a notable surge in interest surrounding pyrazoline as a promising heterocyclic framework within the realm of medicine. This heightened attention can be attributed to the widespread use of pyrazoline in the development of therapeutic medications. The compound has a significant role in various fields, such as synthetic and medicinal chemistry, serving as a crucial foundational component for the production of pharmaceuticals. Heterocyclic compounds have been found to have an affinity towards a diverse range of biotargets across several fields [27,28,29]. Both agribusiness and pharmacological research have derived advantages from the utilization of these scaffolds [30].

There have been numerous reports of thiazole and pyrazoline scaffolds exhibiting a broad range of chemical reactivity and pharmacological activities for decades [31,32]. Numerous studies have demonstrated that these five-membered heterocyclic compounds delocalize free radicals and inhibit bacteria by producing stable DPPH fragments [33]. Thiazole-based pyrazoline scaffolds also exhibit anti-inflammatory [34], antidiabetic [35], Alzheimer’s disease [36], anti-HIV [37], antiviral [38], anticonvulsant, antitumor activities [39], and antimicrobial properties [40]. The pyrazoline skeleton has been extensively studied using a variety of structural manipulations and it has been concluded that the steric and electronic properties of substitutes at the N-1, C-3, and C-5 positions, as well as their chirality, have significant effects on their biological activity [41]. A pyrazoline’s N-N bonds are said to play an important role in its pharmacological properties [42].

Pyrazoline structures have been found to possess noteworthy pharmacological properties, encompassing anti-inflammatory, antioxidant, analgesic, antifungal, antipyretic, antiangiogenic, antibacterial, antitumor, and antiviral activities [43,44]. Our research group is presently involved in the development of novel and more effective synthetic routes for small compounds. This endeavour aims to enable the investigation of their potential biological activities, specifically their inhibitory effects on enzymes such as α-glucosidase and urease, which have been observed to be significant.

**Figure 1:**
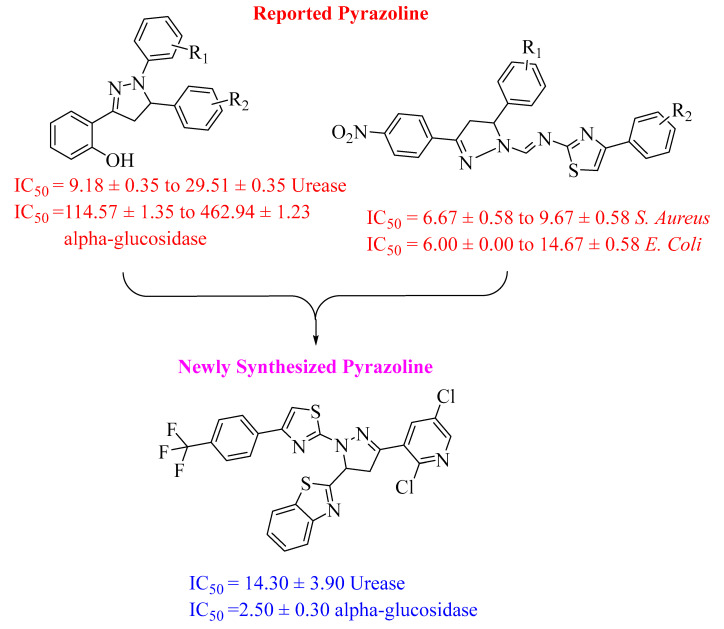
Rational study of the synthesized pyrazoline-based thiazole analogues.

As pyrazoline structural patterns play an important role in medicinal chemistry, we present our findings concerning the synthesis, characterization, and assessment of novel pyrazoline derivatives. As dual inhibitors, these derivatives inhibit both α-glucosidase and urease enzymes. A variety of pyrazoline derivatives [45,46] containing different substitution arrangements were evaluated for their ability to inhibit both *α*-glucosidase and urease enzymes. Among heterocyclic compounds, these analogues have shown a considerable potency, providing a greater pool of potential of α-glucosidase and urease inhibitors than previously known (Figure 1).

## 2. Results and Discussion

### 2.1. Chemistry

**Scheme 1:**
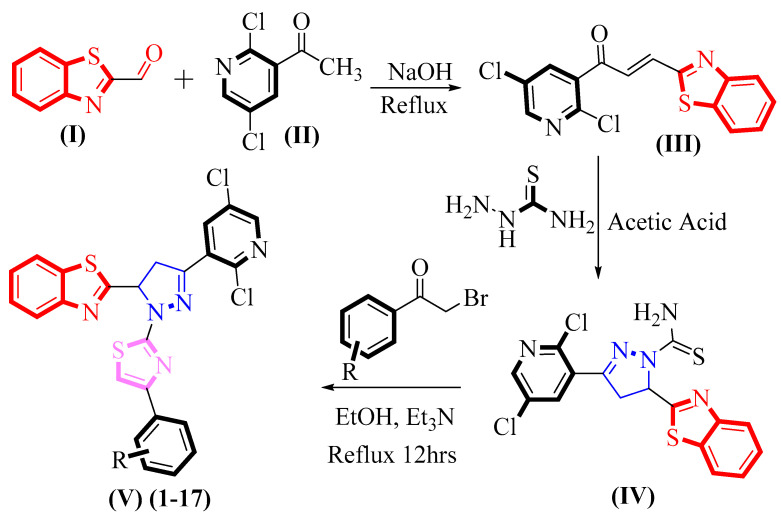
General scheme for the synthesis of benzothiazole-derived pyrazoline-based thiazole (**1**–**17**).

In order to synthesize pyrazoline-based thiazoles, a multi-step reaction procedure was involved. The desired pyrazoline-based thiazoles derivatives (**1−17**) were synthesized using the method outlined in Scheme 1. In the first step, 1-(2,5-dichloropyridin-3-yl)ethan-1-one (**I**) was reacted with benzo[d]thiazole-2-carbaldehyde (**II**) to produce a substrate chalcone (**III**) [47]. This reaction was carried out in the presence of a 50% sodium hydroxide solution in absolute ethanol. It was refluxed for 8–10 h continuously with constant stirring in an ice bath. The substrate (**III**) was neutralized with dilute hydrochloric acid and washed with pet-ether. The substrate (**III**) endured cyclization by reacting with thiosemicarbazide that was stirred in acetic acid, and heated under reflux conditions for 5–7 h, enabling the formation of an intermediate pyrazoline (**IV**). Finally, the substrate (**IV**) was reacted with differently substituted phenacyl bromide in the presence of triethyl amine (Et_3_N) in solvent absolute ethanol, affording the targeted pyrazoline-based thiazole derivatives (**V**) in an appropriate yield (Scheme 1).

### 2.2. Biological Activities

#### 2.2.1. In Vitro α-Glucosidase and Urease Inhibitory Potential

The synthesized compounds of the benzothiazole-derived pyrazoline-based thiazole skeleton (**1**–**17**) were subjected to *α*-glucosidase and urease inhibitory activities. All the newly afforded compounds showed *α*-glucosidase and urease inhibitory potential in the range of IC_50_ = 2.50 ± 0.30–17.50 ± 0.20 µM and 14.30 ± 3.90–41.50 ± 1.70 µM, respectively. The obtained results were compared to those of the standard drug acarbose (IC_50_ = 5.30 ± 0.30 µM) and thiourea drug (IC_50_ = 31.40 ± 2.50 µM). It is noteworthy that, among the synthesized analogues, the analogues **6** (IC_50_ = 2.50 ± 0.30 µM) and (IC_50_ = 14.30 ± 3.90 µM), analogue **7** (IC_50_ = 3.20 ± 0.10 µM) and (IC_50_ = 19.20 ± 0.10 µM), and analogue 12 (IC_50_ = 3.50 ± 0.10 µM) and (IC_50_ = 22.30 ± 0.80 µM) showed more significant potency, as well as being manifold more active than the standard drug acarbose and thiourea. Moreover, the remaining analogues demonstrated moderate and low *α-*glucosidase and urease inhibitory activities. However, the substitution pattern −CF_3_, −F, and −OH were the crucial factors for the enzyme inhibition activities of the synthesized analogues. Based on the existence of the −CF_3_ electron-withdrawing activity on the phenyl ring, it was determined that compound **6** is a dual powerful inhibitor against *α*-glucosidase and urease among the synthesized benzothiazole-derived pyrazoline-based thiazole series (**1**–**17**).

#### 2.2.2. Structure–Activity Relationship (SAR) for α-Glucosidase and Urease Inhibitory

The in vitro inhibitory activities against α-glucosidase and urease were examined for all the synthetic analogues of the pyrazoline-based thiazole compounds (**1**–**17**). In-depth structure–activity relationship (SAR) investigations were carried out, primarily focusing on the central core of the pyrazoline-based thiazole scaffold and its various substitution patterns. These findings suggest that distinct substituents were responsible for the observed differences in the *α*-glucosidase and urease inhibitory activities. These biochemical activities were influenced by only a limited number of substitution patterns on the pyrazoline scaffold.

It seemed from the SAR analysis that analogues **6** (IC_50_ = 2.50 ± 0.30 µM, IC_50_ = 14.30 ± 3.90 µM), **7** (IC_50_ = 3.20 ± 0.10 µM, IC_50_ = 19.20 ± 0.10 µM), **14** (IC_50_ = 3.40 ± 0.10 µM, IC_50_ = 21.80 ± 2.90 µM), and **12** (IC_50_ = 3.50 ± 0.10 µM, IC_50_ = 22.30 ± 0.80 µM) were identified as potent inhibitors of targeted α-glucosidase and urease enzymes, even demonstrating more potency when compared to the standard acarbose (IC_50_ = 5.30 ± 0.30 µM) and thiourea (IC_50_ = 31.40 ± 2.50 µM) drugs, respectively.

Compound **6**, with an IC_50_ of 2.50 ± 0.30 M for *α*-glucosidase and IC_50_ = 14.30 ± 3.90 µM for urease inhibition, demonstrated an impressive profile, which was partly attributed to its trifluoromethyl group [48]. Trifluoromethyl groups have a strong affinity for the enzyme’s active sites, primarily through hydrogen bonding interactions. Additionally, the -CF_3_ group reduces the electronic density of the aromatic ring, increasing its acidity and affecting the electrostatic interactions with the substrate. This accounts for the compound’s significant inhibitory activities against *α*-glucosidase and urease. Furthermore, compound **7** (IC_50_ = 3.20 ± 0.10 µM and IC_50_ = 19.20 ± 0.10 µM) contains *para*-fluoro and *ortho*-hydroxy groups, while compound **12** (IC_50_ = 3.50 ± 0.10 µM and IC_50_ = 22.30 ± 0.80 µM) features a *para*-fluoro group attached to the aromatic ring. Due to the nucleophilicity, metabolic stability, and hydrogen bonding capabilities of the hydroxy and fluoro groups, these structural features enhance the enzyme inhibitory potential. Thus, these compounds have notable *α*-glucosidase and urease inhibitory properties. Furthermore, compounds **11** and **12**, which both contain fluorine in *meta*- and *para*-positions, enhance their biological potential via their elevated electronegativity and ability to form hydrogen bonds with enzyme active sites. Compound **12** (IC_50_ = 3.50 ± 0.10 µM and 22.30 ± 0.80µM), with a *para*-fluoro group, exhibits a higher inhibitory potential compared to compound **11** (IC_50_ = 3.80 ± 0.30 µM and 25.30 ± 0.20µM), which has a *meta*-fluoro group. Due to the presence of *meta*-fluoro, the inhibitory potential may be decreased. Consequently, substituents play a significant role in determining *α*-glucosidase and urease inhibitory profiles (Table 1).

Compounds **14** (IC_50_ = 3.40 ± 0.10 µM and 21.80 ± 2.90), **9** (IC_50_ = 4.40 ± 0.30 µM and 29.40 ± 0.10), and **17** (IC_50_ = 6.80 ± 0.20 µM and 31.30 ± 0.40) exhibited notably higher *α*-glucosidase and urease inhibitory potential. The enhanced activity is attributed to the presence of an *ortho*-hydroxy group attached to the aromatic ring, as well as a di-chloro group in the *ortho-meta* position. By increasing the binding affinity with the substrate and forming hydrogen bonds with the enzyme’s active sites, the hydroxy group plays a crucial role in the inhibitory profile. Furthermore, the presence of two chlorine groups that are electron-donating for a resonance effect makes the hydroxyl group much more willing to form hydrogen bonds with the biological target. Further, compound **9** demonstrated a higher potency than compound **17**, primarily due to the presence of a nitro group, which enhances the inhibitory potential of the scaffolds. As a toxicophore and a pharmacophore, the nitro group induces charge density by withdrawing electrons from the aromatic system. The -Cl group also influences the electronic cloud through an inductive effect on the aromatic system. As a result, the presence of -NO_2_ and -Cl groups significantly enhances the biological profile of the molecule. As a result of the *ortho–meta* interaction within the aromatic system, compound **14** exhibited a superior inhibitory potential compared to both **9** and **17**.The position of substituents on the phenyl ring of the pyrazoline–thiazole skeleton plays a crucial role in determining the *α*-glucosidase and urease inhibitory potential shown below in Table 1.

Compound **4** (IC_50_ = 4.10 ± 0.30 µM and 23.30 ± 2.40) possesses a *para*-nitro group that exhibits strong binding with enzyme active sites, resulting in an increase in potency. The electron-withdrawing properties of nitro groups cause charge density, which is explained by the nitro group’s electron-withdrawing properties. However, compound 1 (IC_50_ = 13.10 ± 0.40 µM and 41.50 ± 1.70) has a *para*-bromo group and an *ortho*-nitro group, with the *para*-bromo group causing steric hindrance, resulting in a reduced potency in comparison to compound **4**, as well as acarbose and thiourea (IC_50_ = 5.30 ± 0.30 µM and IC_50_ = 31.40 ± 2.50 µM, respectively). A substituent at the *ortho*-nitro position of compound 5 (IC_50_ = 6.70 ± 0.30 µM and 32.50 ± 2.10) also exhibited a lower potency than **4** because of the position of the substituent of the -NO_2_ group on the *meta-*position. Hence, these scaffolds have different inhibitory potentials against alpha-glucosidase and urease depending on the position of the substituent, as shown in Table 1.

Additionally, compounds **8** and **13** (IC_50_ = 13.10 ± 0.10 µM and 39.20 ± 0.60), **2** (IC_50_ = 14.20 ± 0.10 µM and 37.20 ± 1.90), and **16** (N.A.) exhibited poor inhibitory activity due to the presence of bulky groups such as N,N-dimethyl, *ortho-meta*-dimethyl, benzyloxy-phenyl, and naphthalene, which cause steric hindrance. These compounds are less potent when compared to the standard drugs acarbose and thiourea (IC_50_ = 5.30 ± 0.30 µM and IC_50_ = 31.40 ± 2.50 µM, respectively). Accordingly, bulky groups lead to a lower efficacy as compared to standard drugs. As such, the biological significance of scaffolds can also vary depending on the position and nature of their substituents, either through hydrogen bonding or Van der Waals interactions. Accordingly, the synthesized analogues have electron-withdrawing properties (-CF_3_, -NO_2_, and -Cl) and strong hydrogen bonding substituents, such as the -OH and -F groups, that make them excellent α-glucosidase and urease inhibitors. Accordingly, the inhibitory profile of the synthesized compounds depends on the position, nature, and number of substituents attached to the aromatic ring of the benzothiazole-derived pyrazoline-based thiazole moiety (**1**–**17**).

### 2.3. Molecular Docking Studies

**Figure 2:**
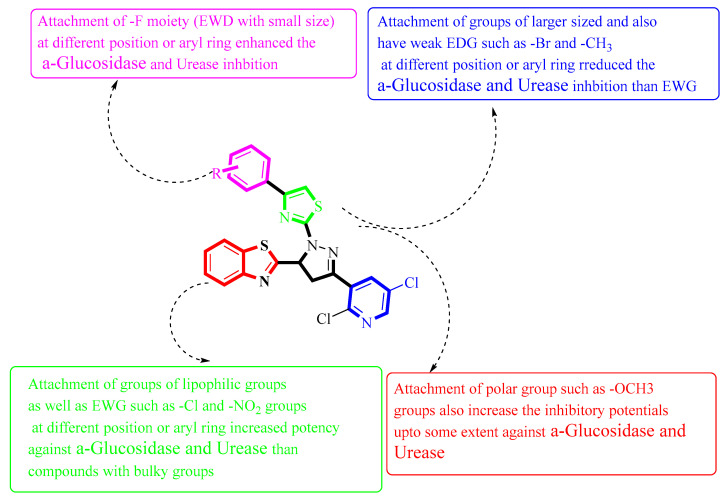
Summary of SAR studies of pyrazoline–thiazole hybrids analogues against ***α***–glucosidase and urease enzymes.

**Figure 3:**
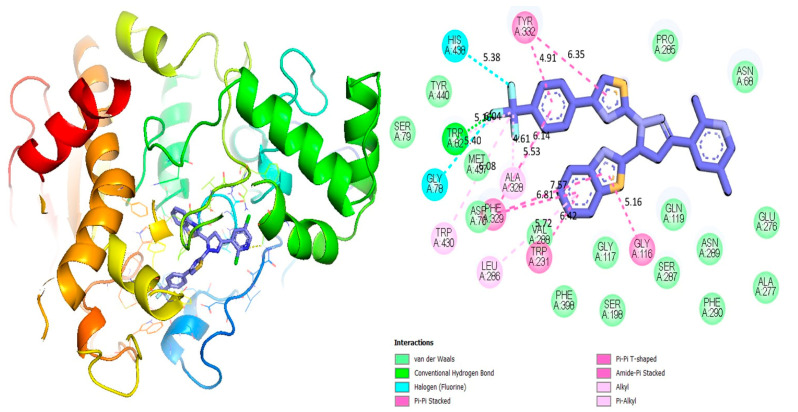
Protein–ligand interaction 2D and 3D profile of an active analogue **6** against α-glucosidase.

**Figure 4:**
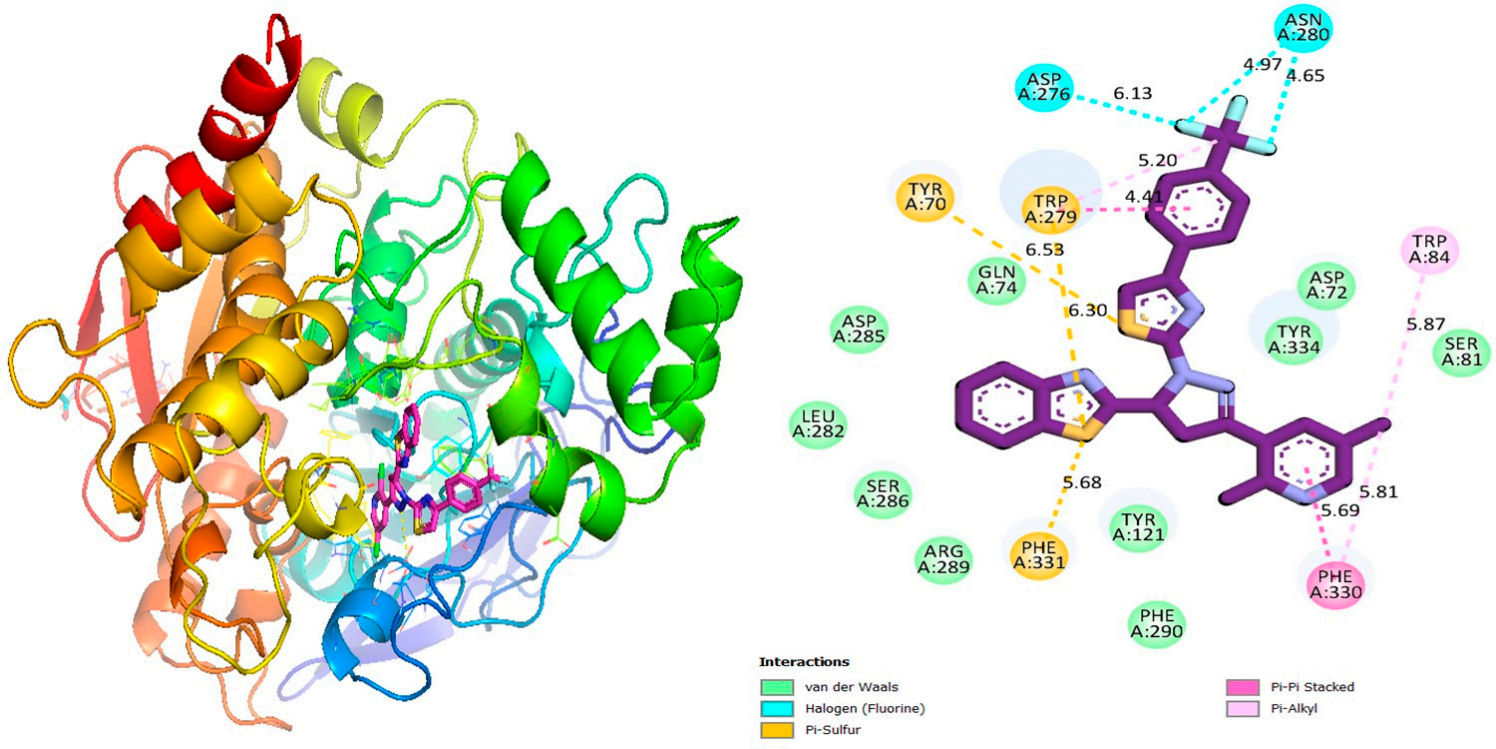
Protein–ligand interaction 2D and 3D profile of an active analogue **6** against urease.

**Figure 5:**
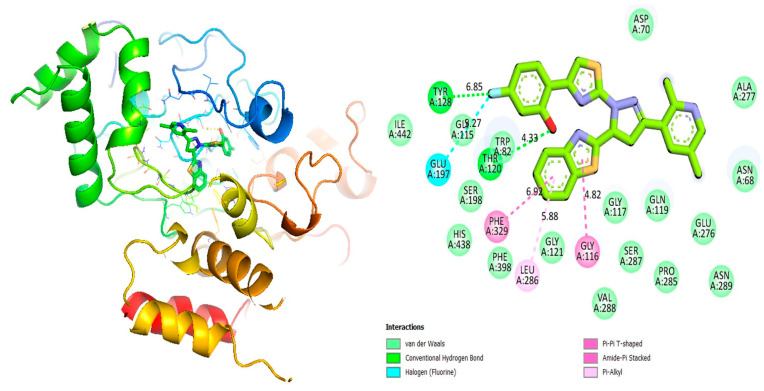
Protein–ligand interaction 2D and 3D profile of an active analogue **7** against α-glucosidase.

**Figure 6:**
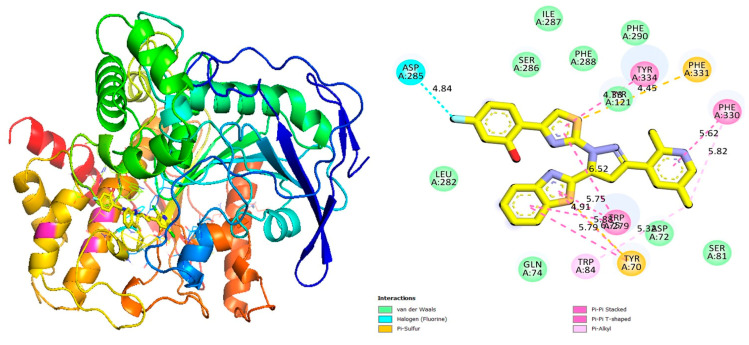
Protein–ligand interaction 2D and 3D profile of an active analogue **7** against urease.

**Figure 7:**
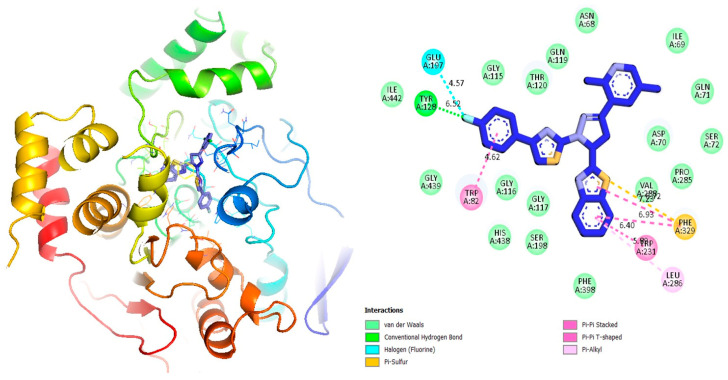
Protein–ligand interaction 2D and 3D profile of an active analogue **12** against α-glucosidase.

**Figure 8:**
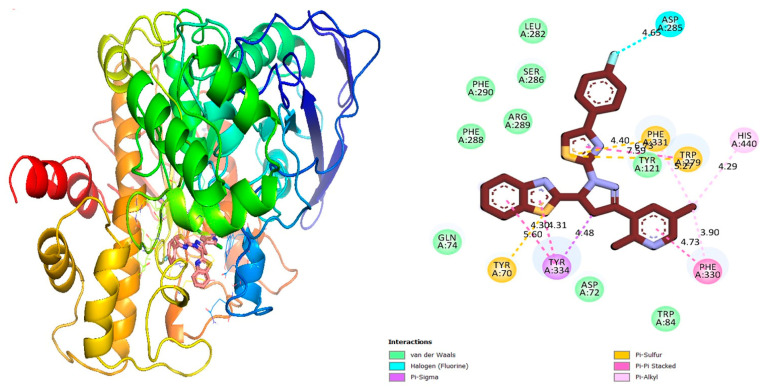
Protein–ligand interaction 2D and 3D profile of an active analogue **12** urease.

**Figure 9:**
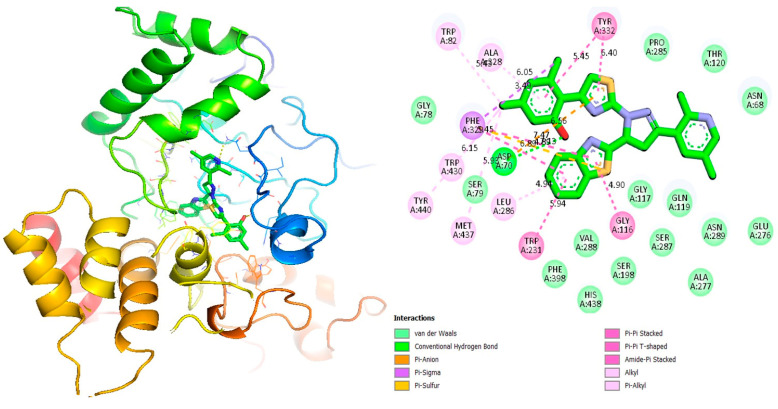
Protein–ligand interaction 2D and 3D profile of an active analogue **14** against α-glucosidase.

**Figure 10:**
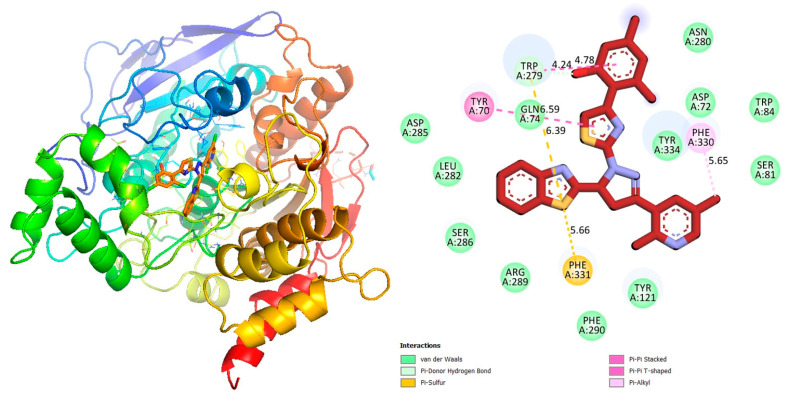
Protein–ligand interaction 2D and 3D profile of an active analogue **7** against urease.

**Figure 11:**
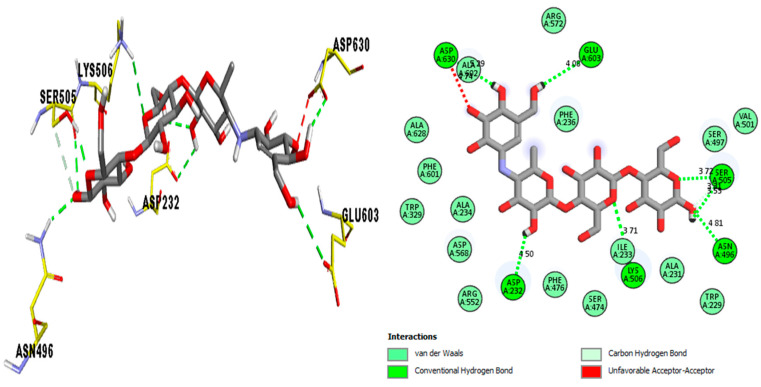
Protein–ligand interaction 2D and 3D profile of acarbose against α-glucosidase.

**Figure 12:**
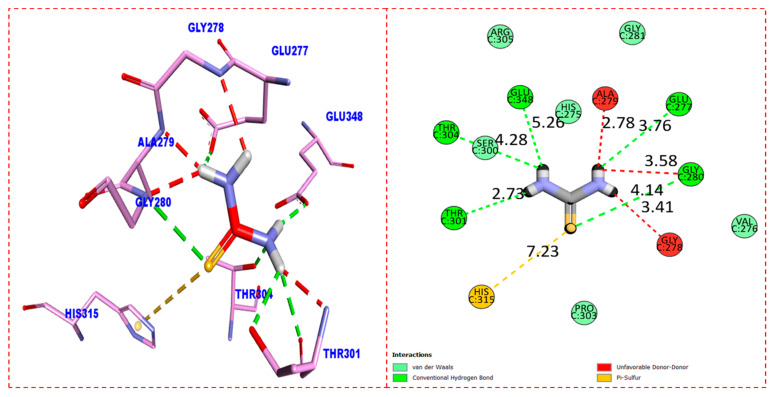
Protein–ligand interaction 2D and 3D profile of thiourea against urease.

In this study, docking studies enable us to identify robust binding interactions between specific ligands and enzyme active sites. The investigation involved various substitutions in pyrazoline-based thiazole scaffolds (**1**–**17**). These compounds were shown to be effective against the targeted proteins through molecular docking analyses. As a result of using a variety of software tools, we were able to visualize the interaction between ligands and amino acids, determining their distances and interaction types, such as C-H bonds, pi-R interactions, pi-S interactions, hydrogen bonds, pi-pi T-shaped interactions, pi-stacking, and pi-sigma interactions, which are shown below in Figure 2, Figure 3, Figure 4, Figure 5, Figure 6, Figure 7, Figure 8, Figure 9, Figure 10, Figure 11 and Figure 12 and Table 2. Our research team synthesized various heterocyclic compounds with a variety of substitutions and evaluated their potential biological activity using diverse molecular docking software [48,49,50,51,52,53].

## 3. Materials and Methods 

### 3.1. General Information 

This study used chemicals and solvents that were obtained from reputable suppliers, namely Merck and Sigma-Aldrich, without any further purification. An electrothermal digital device was used to determine the melting points. A Bio-Rad spectrophotometer was used to acquire the infrared spectra. Bruker spectrometers with proton frequencies of 600 MHz and carbon frequencies of 126 MHz were used to acquire the NMR spectra. Nuclear magnetic resonance (NMR) chemical shift values were first established using the δ (parts per million) scale. Thin-layer chromatography (TLC) was employed as a means of assessing and overseeing the advancement and culmination of the reaction. The spots on the TLC plate were visualized by exposing it to 254 nm ultraviolet light (UV). In addition to DMSO-*d_6_*, a reference compound was used to calculate chemical shift values. Moreover, the coupling constants are expressed in hertz (Hz). We acquired mass spectra using the mass spectrometers MAT 113D, JEOL JMS-600H, and MAT 312 (high-resolution electron ionization mass spectrometry (HR-EI-MS).

### 3.2. General Procedure for the Synthesis of Chalcone

#### 3.2.1. Synthesis of Chalcone Derivatives

In the first step, chalcone was synthesized using the Claisen–Schmidt condensation reaction, in which 1 mmol of 1-(2,5-dichloropyridin-3-yl)ethan-1-one was taken in a round-bottom flask and sodium hydroxide (40% NaOH) was added as a catalyst in solvent ethanol and stirred in the solution for 30 min in an ice bath, followed by the addition of 1 mmol of benzo[d]thiazole-2-carbaldehyde dropwise into it. The reaction was refluxed and stirred for from 8 to 10 h to form targeted product chalcone derivatives in a good yield. The obtained product was neutralized with dilute hydrochloric acid (10%), washed with chilled pet-ether and *n*-hexane, recrystallized from ethanol, and then dried as final products.

#### 3.2.2. Synthesis of Pyrazoline

In this step, chalcone (1 mmol) was reacted with 1 mmol of thiosemicarbazide in the presence of 5 mL of acetic acid in ethanol. The reaction was refluxed for 7–10 h, yielding pyrazoline analogues. The reaction progress was monitored through TLC. The product was washed with chilled pet-ether, recrystallized with ethanol, and dried as a final product for further reaction.

#### 3.2.3. Synthesis of Pyrazoline Derivatives with Phenacyl Bromide

In the last step, the 1 mmol of pyrazoline substrate was reacted with 1 mmol of differently substituted phenacyl bromide in the presence of triethyl amine (2–4 drops) in solvent absolute ethanol, affording the targeted benzothiazole-derived pyrazoline-based thiazole derivatives in an appropriate yield. The progress of the reaction was monitored through TLC. The solvent was evaporated through a rotary evaporator. The synthesized products were washed with *n*-hexane and recrystallized in chloroform, yielding pure products. The synthesized product was characterized through different spectroscopic techniques, including ^1^H-NMR, ^13^C-NMR, and HREI-MS (Scheme 1).

### 3.3. Spectral Analysis

#### 3.3.1. 2-(1-(4-(4-Bromo-2-nitrophenyl)thiazol-2-yl)-3-(2,5-dichloropyridin-3-yl)-4,5-dihydro-1H-pyrazol-5-yl)benzo[d]thiazole (**1**)

Physical state: solid; *Rf* value: 0.68 (4:6, *n*-Hex:EtOAc); Melting Point: 233–234 °C; Colour: light-yellow; Yield = 45%, FT-IR (cm^−1^): 3041 (C=CHstr), 2869 (CHstr), 1676 (C=Nimine), 1540 (C-NO_2_), 1620 (C=Cstr), 1421 (C=Cbend), 1187 (C-N), 654 (C-Br), 584 (C-Cl); ^1^H-NMR (600 MHz, DMSO-*d6*): *δ* 9.17 (s, 1H, pyridin-H), 9.02 (s, 1H, pyridin-H), 8.87 (s, 1H, Ar-H), 8.41 (dd, *J* = 8.6, 2.4 Hz, 1H, Benzothiazol-H), 8.29 (dd, *J*= 8.9, 2.6 Hz, 1H, Benzothiazol-H), 8.26 (d, *J* = 8.0 Hz, 1H, Ar-H), 8.17 (d, *J* = 8.2 Hz, 1H, Ar-H), 7.75 (t, *J* = 8.0 Hz, 2H, Benzothiazol-H), 7.56 (s, 1H, thiazol-H), 4.90 (dd, 1H, pyrazoline-H), 3.86 (dd, 1H, pyrazoline-H), 2.96 (dd, 1H, pyrazoline-H); ^13^C-NMR (150 MHz, DMSO-*d6*): *δ* 170.1, 155.6, 154.7, 150.1, 147.8, 145.6, 144.1, 143.0, 142.5, 141.8, 140.4, 139.5, 139.3, 138.7, 138.5, 137.2, 134.0, 132.3, 127.6, 125.4, 121.3, 109.6, 64.9, 40.4; HREI-MS: *m/z* calcld for C_24_H_13_Cl_2_BrN_6_O_2_S_2_, [M]+ 632.1357 Found 632.1348.

#### 3.3.2. 2-(3-(2,5-Dichloropyridin-3-yl)-1-(4-(2,5-dimethylphenyl)thiazol-2-yl)-4,5-dihydro-1H-pyrazol-5-yl)benzo[d]thiazole (**2**)

Physical state: solid; *Rf* value: 0.71 (4:6, *n*-Hex:EtOAc); Melting Point: 219–220 °C; Colour: light-yellow; Yield= 49%,FT-IR (cm^−1^): 3085 (C=CHstr), 2831 (CHstr), 1656 (C=Nimine), 1627 (C=Cstr), 1453 (C=Cbend), 1140 (C-N), 576 (C-Cl); ^1^H-NMR (600 MHz, DMSO-d6): *δ* 9.11 (s, 1H, pyridin-H), 9.04 (s, 1H, pyridin-H), 8.39 (dd, *J* = 8.0, 1.6 Hz, 1H, Benzothiazol-H), 8.31 (dd, *J* = 8.7, 1.6 Hz, 1H, Benzothiazol-H), 8.25 (s, 1H, Ar-H), 8.19 (d, *J* = 8.7 Hz, 1H, Ar-H), 7.92 (d, 1H, *J* = 8.2 Hz, Ar-H), 7.79 (t, *J* = 8.0 Hz, 2H, Benzothiazol-H), 7.59 (s, 1H, thiazol-H), 4.93 (dd, 1H, pyrazoline-H), 3.82 (dd, 1H, pyrazoline-H), 2.91 (dd, 1H, pyrazoline-H), 2.65 (s, 6H, Ar-CH_3_); ^13^C-NMR (150 MHz, DMSO-d6): *δ* 173.5, 154.7, 153.5, 153.2, 144.0, 143.4, 142.4, 141.5, 138.7, 136.2, 134.8, 134.2, 130.2, 130.0, 139.5, 128.4, 128.3, 125.2, 115.0, 113.3, 112.5, 102.1, 59.7, 43.8, 32.8, 31.5; HREI-MS: *m/z* calcld for C_26_H_19_Cl_2_N_5_S_2_, [M]+ 536.4365 Found 536.4336. 

#### 3.3.3. 2-(3-(2,5-Dichloropyridin-3-yl)-1-(4-(p-tolyl)thiazol-2-yl)-4,5-dihydro-1H-pyrazol-5-yl)benzo[d]thiazole (**3**)

Physical state: solid; *Rf* value: 0.47 (7:3, *n*-Hex:EtOAc); Melting Point: 227–228 °C; Colour: off-white;Yield= 43%, FT-IR (cm^−1^): 3043 (C=CHstr), 2867 (CHstr), 1665 (C=Nimine), 1619 (C=Cstr), 1439 (C=Cbend), 1161 (C-N), 604 (C-Cl); ^1^H-NMR (600 MHz, DMSO-*d6*): *δ* 9.14 (s, 1H, pyridin-H), 9.05 (s, 1H, pyridin-H), 8.43 (dd, *J* = 8.5, 2.0 Hz, 1H, Benzothiazol-H), 8.36 (dd, *J* = 8.0, 1.9 Hz, 1H, Benzothiazol-H), 8.21 (d*, J* = 8.4 Hz, 2H, Ar-H), 8.04 (d, 2H, *J* = 8.0 Hz,Ar-H), 7.77 (t, J = 8.0 Hz, 2H, Benzothiazol-H), 7.63 (s, 1H, thiazol-H), 4.89 (dd, 1H, pyrazoline-H), 3.87 (dd, 1H, pyrazoline-H), 2.93 (dd, 1H, pyrazoline-H), 2.61 (s, 3H, Ar-CH_3_); ^13^C-NMR (150 MHz, DMSO-*d6*): δ 174.6, 157.2, 156.4, 156.2, 153.9, 153.5, 152.4, 150.1, 150.0, 147.5, 148.7, 147.0, 143.2, 143.1, 139.3, 138.8, 132.4, 123.5, 121.7, 117.4, 112.8, 101.2, 59.7, 45.3, 32.4; HREI-MS: *m/z* calcld for C_25_H_17_Cl_2_N_5_S_2_, [M]+ 522.4743 Found 522.4704. 

#### 3.3.4. 2-(3-(2,5-Dichloropyridin-3-yl)-1-(4-(4-nitrophenyl)thiazol-2-yl)-4,5-dihydro-1H-pyrazol-5-yl)benzo[d]thiazole (**4**)

Physical state: solid; *Rf* value: 0.58 (5:5, *n*-Hex:EtOAc); Melting Point: 232–233 °C; Colour: dirty-white;Yield= 61%, FT-IR (cm^−1^): 3065 (C=CHstr), 2857 (CHstr), 1644 (C=Nimine), 1610 (C=Cstr), 1534 (C-NO_2_), 1442 (C=Cbend), 1149 (C-N), 591 (C-Cl); ^1^H-NMR (600 MHz, DMSO-d6): δ 9.17 (s, 1H, pyridin-H), 9.15 (s, 1H, pyridin-H), 8.56 (dd, *J* = 8.0, 2.2 Hz, 1H, benzothiazol-H), 8.43 (dd, *J* = 8.3, 2.5 Hz, 1H, benzothiazol-H), 8.37 (d, *J* = 8.2 Hz, 2H, Ar-H), 8.16 (d, 2H, *J* = 8.3 Hz, Ar-H), 8.02 (t, *J* = 7.7 Hz, 2H, benzothiazol-H), 7.88 (s, 1H, thiazol-H), 4.81 (dd, 1H, pyrazoline-H), 3.92 (dd, 1H, pyrazoline-H), 2.88 (dd, 1H, pyrazoline-H); ^13^C-NMR (150 MHz, DMSO-*d6*): δ 174.2, 154.5, 154.4, 152.9, 150.3, 145.0, 143.6, 139.3, 137.2, 136.4, 135.8, 132.5, 131.9, 131.6, 129.9, 129.4, 128.0, 127.2, 126.1, 124.5, 119.4, 103.9, 59.9, 43.7; HREI-MS: *m/z* calcld for C_24_H_14_Cl_2_N_6_S_2_O_2_, [M]+ 553.1534 Found 553.1504.

#### 3.3.5. 2-(3-(2,5-Dichloropyridin-3-yl)-1-(4-(2-nitrophenyl)thiazol-2-yl)-4,5-dihydro-1H-pyrazol-5-yl)benzo[d]thiazole (**5**)

Physical state: solid; *Rf* value: 0.70 (4:6, *n*-Hex:EtOAc); Melting Point: 224–225 °C; Colour: off-white; Yield = 53%, FT-IR (cm^−1^): 3074 (C=CHstr), 2838 (CHstr), 1652 (C=Nimine), 1623 (C=Cstr), 1537 (C-NO_2_), 1435 (C=Cbend), 1131 (C-N), 593 (C-Cl); ^1^H-NMR (600 MHz, DMSO-*d6*): δ 9.15 (s, 1H, pyridin-H), 9.08 (s, 1H, pyridin-H), 8.67 (dd, 1H, *J* = 7.4, 1.3 Hz, Ar-H), 8.45 (dd, *J* = 8.0, 2.2 Hz, 1H, Benzothiazol-H), 8.39 (dd, *J* = 8.2, 1.6 Hz, 1H, Benzothiazol-H), 8.33 (dd, *J* = 7.9, 2.0 Hz, 1H, Ar-H), 8.27 (t, *J* = 7.6 Hz, 1H, Ar-H), 7.95 (t, *J* = 7.6 Hz, 2H, Benzothiazol-H), 7.87 (t, *J* = 7.4 Hz, 1H, Ar-H), 7.55 (s, 1H, thiazol-H), 4.81 (dd, 1H, pyrazoline-H), 3.77 (dd, 1H, pyrazoline-H), 2.86 (dd, 1H, pyrazoline-H); ^13^C-NMR (150 MHz, DMSO-*d6*): δ 173.4, 156.5, 156.4, 154.0, 151.7, 148.9, 147.2, 144.6, 143.7, 140.2, 139.9, 137.4, 132.8, 130.8, 129.9, 126.4, 123.9, 120.2, 129.8, 124.5, 120.5, 100.5, 58.6, 43.4; HREI-MS: *m/z* calcld for C_24_H_14_Cl_2_N_6_O_2_S_2_, [M]+ 553.2652 Found 553.2631.

#### 3.3.6. 2-(3-(2,5-Dichloropyridin-3-yl)-1-(4-(4-(trifluoromethyl)phenyl)thiazol-2-yl)-4,5-dihydro-1H-pyrazol-5-yl)benzo[d]thiazole (**6**)

Physical state: solid; *Rf* value: 0.65 (6:4, *n*-Hex:EtOAc); Melting Point: 225–226 °C; Colour: colourless; Yield = 57%, FT-IR (cm^−1^): 3057 (C=CHstr), 2892 (CHstr), 1673 (C=Nimine), 1649 (C=Cstr), 1465 (C=Cbend), 1187 (C-N), 1087 (C-F), 612 (C-Cl); ^1^H-NMR (600 MHz, DMSO-*d6*): δ 9.16 (s, 1H, pyridin-H), 9.11 (s, 1H, pyridin-H), 8.63 (dd, *J* = 8.2, 2.0 Hz, 1H, benzothiazol-H), 8.57 (dd, *J* = 7.2, 2.7 Hz, 1H, benzothiazol-H), 8.44 (d, *J* = 8.0 Hz, 2H, Ar-H), 8.32 (d, 2H, *J* = 8.1 Hz, Ar-H), 8.16 (t, *J* = 7.9 Hz, 2H, benzothiazol-H), 7.91 (s, 1H, thiazol-H), 4.82 (dd, 1H, pyrazoline-H), 3.91 (dd, 1H, pyrazoline-H), 2.85 (dd, 1H, pyrazoline-H); ^13^C-NMR (150 MHz, DMSO-*d6*): δ 175.9, 156.4, 155.4, 154.3, 150.7, 146.0, 145.9, 143.1, 140.7, 139.2, 137.9, 137.6, 136.7, 134.5, 131.2, 129.1, 128.4, 126.3, 122.2, 121.0, 120.4, 116.4, 112.2, 57.4, 40.3; HREI-MS: *m/z* calcld for C_25_H_14_Cl_2_F_3_N_5_S_2_, [M]+ 576.9576 Found 576.9558.

#### 3.3.7. 2-(2-(5-(Benzo[d]thiazol-2-yl)-3-(2,5-dichloropyridin-3-yl)-4,5-dihydro-1H-pyrazol-1-yl)thiazol-4-yl)-5-fluorophenol (**7**)

Physical state: solid; *Rf* value: 0.61 (4:6, *n*-Hex:EtOAc); Melting Point: 228–229 °C; Colour: light brown; Yield = 56%, FT-IR (cm^−1^): 3286 (-OH), 3064 (C=CHstr), 2873 (CHstr), 1687 (C=Nimine), 1632 (C=Cstr), 1456 (C=Cbend), 1205 (C-N), 1077 (C-F), 598 (C-Cl); ^1^H-NMR (600 MHz, DMSO-*d6*): δ 10.02 (s, 1H, -OH), 9.08 (s, 1H, pyridin-H), 8.99 (s, 1H, pyridin-H), 8.66 (dd, *J* = 8.0, 2.2 Hz, 1H, Benzothiazol-H), 8.47 (dd, *J* = 8.2, 1.9 Hz, 1H, Benzothiazol-H), 8.26 (d, *J* = 7.0 Hz, 1H, Ar-H), 8.17 (t, *J* = 8.3 Hz, 2H, Benzothiazol-H), 8.12 (d, *J* = 7.2 Hz, 1H, Ar-H), 7.93 (s, 1H, Ar-H), 7.56 (s, 1H, thiazol-H), 4.85 (dd, 1H, pyrazoline-H), 3.79 (dd, 1H, pyrazoline-H), 2.88 (dd, 1H, pyrazoline-H); ^13^C-NMR (150 MHz, DMSO-*d6*): *δ* 175.2, 155.2, 153.4, 153.2, 151.4, 146.6, 144.9, 143.8, 139.8, 137.8, 135.0, 134.5, 133.2, 132.6, 132.2, 131.8, 128.0, 126.4, 124.8, 120.6, 112.4, 103.6, 59.9, 43.4; HREI-MS: *m/z* calcld for C_24_H_14_Cl_2_FN_5_OS_2_, [M]+ 542.4376 Found 542.4352.

#### 3.3.8. 4-(2-(5-(Benzo[d]thiazol-2-yl)-3-(2,5-dichloropyridin-3-yl)-4,5-dihydro-1H-pyrazol-1-yl)thiazol-4-yl)-N,N-dimethylaniline (**8**)

Physical state: solid; *Rf* value: 0.49 (7:3, *n*-Hex:EtOAc); Melting Point: 231–232 °C; Colour: light yellow; Yield = 60%, FT-IR (cm^−1^): 3082 (C=CHstr), 2898 (CHstr), 1676 (C=Nimine), 1627 (C=Cstr), 1464 (C=Cbend), 1204 (C-N), 611 (C-Cl); ^1^H-NMR (600 MHz, DMSO-*d6*): δ 9.12 (s, 1H, pyridin-H), 9.09 (s, 1H, pyridin-H), 8.56 (dd, *J* = 8.1, 2.3 Hz, 1H, benzothiazol-H), 8.49 (dd, *J* = 7.8, 2.3 Hz, 1H, benzothiazol-H), 8.37 (d, *J* = 8.0 Hz, 2H, Ar-H), 8.33 (d, 2H, *J* = 8.2 Hz, Ar-H), 8.19 (t, *J* = 8.0 Hz, 2H, benzothiazol-H), 7.88 (s, 1H, thiazol-H), 4.92 (dd, 1H, pyrazoline-H), 3.93 (dd, 1H, pyrazoline-H), 3.24 (s, 6H, N(CH_3_)_2_), 2.89 (dd, 1H, pyrazoline-H); ^13^C-NMR (150 MHz, DMSO-*d6*): δ 172.3, 156.4, 155.5, 152.2, 152.0, 151.3, 149.2, 148.4, 147.2, 144.7, 139.8, 137.4, 136.7, 133.9, 132.5, 128.2, 128.0, 123.8, 121.1, 120.3, 115.5, 112.2, 57.4, 45.4, 45.0, 42.9; HREI-MS: *m/z* calcld for C_26_H_20_Cl_2_N_6_S_2_, [M]+ 551.6367, Found 551.6328.

#### 3.3.9. 2-(1-(4-(4-Chloro-2-nitrophenyl)thiazol-2-yl)-3-(2,5-dichloropyridin-3-yl)-4,5-dihydro-1H-pyrazol-5-yl)benzo[d]thiazole (**9**)

Physical state: solid; *Rf* value: 0.72 (4:6, *n*-Hex:EtOAc); Melting Point: 230–231 °C; Colour: light yellow; Yield = 54%, FT-IR (cm^−1^): 3091 (C=CHstr), 2884 (CHstr), 1687 (C=Nimine), 1632 (C=Cstr), 1454 (C=Cbend), 1197 (C-N), 604 (C-Cl); ^1^H-NMR (600 MHz, DMSO-*d6*): δ 9.06 (s, 1H, pyridin-H), 8.97 (s, 1H, pyridin-H), 8.61 (s, 1H, Ar-H), 8.54 (dd, *J* = 7.9, 2.0 Hz, 1H, Benzothiazol-H), 8.37 (dd, *J*= 8.0, 1.4 Hz, 1H, Benzothiazol-H), 8.28 (d, *J* = 7.2 Hz, 1H, Ar-H), 8.14 (d, *J* = 7.5 Hz, 1H, Ar-H), 8.04 (t, *J* = 8.0 Hz, 2H, Benzothiazol-H), 7.59 (s, 1H, thiazol-H), 4.88 (dd, 1H, pyrazoline-H), 3.72 (dd, 1H, pyrazoline-H), 2.79 (dd, 1H, pyrazoline-H); ^13^C-NMR (150 MHz, DMSO-*d6*): δ 177.4, 155.3, 154.6, 153.8, 153.3, 150.8, 147.2, 146.8, 142.9, 141.8, 141.2, 137.3, 135.6, 133.5, 133.4, 132.2, 131.7, 126.8, 124.4, 121.2, 118.9, 106.7, 57.7, 41.7; HREI-MS: *m/z* calcld for C_24_H_13_Cl_3_N_6_O_2_S_2_, [M]+ 587.0882 Found 587.0859.

#### 3.3.10. 2-(3-(2,5-Dichloropyridin-3-yl)-1-(4-(2,5-dimethoxyphenyl)thiazol-2-yl)-4,5-dihydro-1H-pyrazol-5-yl)benzo[d]thiazole (**10**)

Physical state: solid; *Rf* value: 0.69 (6:4, *n*-Hex:EtOAc); Melting Point: 234–235 °C; Colour: white; Yield = 47%, FT-IR (cm^−1^): 3067 (C=CHstr), 2880 (CHstr), 1676 (C=Nimine), 1622 (C=Cstr), 1447 (C=Cbend), 1183 (C-N), 589 (C-Cl); ^1^H-NMR (600 MHz, DMSO-*d6*): δ 9.06 (s, 1H, pyridin-H), 8.97 (s, 1H, pyridin-H), 8.61 (s, 1H, Ar-H), 8.54 (dd, *J* = 7.9, 2.0 Hz, 1H, Benzothiazol-H), 8.37 (dd, *J* = 8.0, 1.4 Hz, 1H, Benzothiazol-H), 8.28 (d, *J* = 7.2 Hz, 1H, Ar-H), 8.14 (d, *J* = 7.5 Hz, 1H, Ar-H), 8.04 (t, *J* = 8.0 Hz, 2H, Benzothiazol-H), 7.59 (s, 1H, thiazol-H), 4.88 (dd, 1H, pyrazoline-H), 3.87 (s, 6H, (-OCH_3_)_2_), 3.72 (dd, 1H, pyrazoline-H), 2.79 (dd, 1H, pyrazoline-H); ^13^C-NMR (150 MHz, DMSO-*d6*): δ 176.2, 156.7, 155.3, 154.9, 153.0, 152.4, 151.2, 147.6, 147.2, 146.8, 144.3, 140.8, 137.2, 136.9, 133.7, 132.7, 132.9, 129.9, 128.3, 126.8, 124.6, 102.4, 61.9, 56.4, 55.9, 40.4; HREI-MS: *m/z* calcld for C_26_H_19_Cl_2_N_5_O_2_S_2_, [M]+ 568.1872 Found 568.1849.

#### 3.3.11. 2-(3-(2,5-Dichloropyridin-3-yl)-1-(4-(2-fluorophenyl)thiazol-2-yl)-4,5-dihydro-1H-pyrazol-5-yl)benzo[d]thiazole (**11**)

Physical state: solid; *Rf* value: 0.64 (5:5, *n*-Hex:EtOAc); Melting Point: 221–222 °C; Colour: white; Yield = 63%, FT-IR (cm^−1^): 3058 (C=CHstr), 2883 (CHstr), 1669 (C=Nimine), 1628 (C=Cstr), 1434 (C=Cbend), 1240 (C-F), 1174 (C-N), 587 (C-Cl); ^1^H-NMR (600 MHz, DMSO-d6): δ 9.11 (s, 1H, pyridin-H), 8.89 (s, 1H, pyridin-H), 8.57 (dd, *J* = 7.7, 2.1 Hz, 1H, Benzothiazol-H), 8.43 (dd, *J* = 8.1, 1.8 Hz, 1H, Benzothiazol-H), 8.36 (d, *J* = 7.8 Hz, 1H, Ar-H), 8.21 (s, 1H, Ar-H), 8.19 (d, *J* = 7.9 Hz, 1H, Ar-H), 7.95 (t, *J* = 7.8 Hz, 2H, Benzothiazol-H), 7.64 (s, 1H, thiazol-H), 4.81 (dd, 1H, pyrazoline-H), 3.76 (dd, 1H, pyrazoline-H), 3.17 (s, 6H, -N(CH_3_)_2_), 2.70 (dd, 1H, pyrazoline-H); ^13^C-NMR (150 MHz, DMSO-*d6*): δ 177.9, 153.6, 152.5, 151.3, 150.9, 147.0, 146.0, 144.8, 144.7, 144.1, 143.9, 142.6, 139.8, 137.8, 137.2, 135.8, 132.3, 129.5, 129.4, 126.4, 123.7, 103.4, 62.5, 45.2, 44.8, 40.7; HREI-MS: *m/z* calcld for C_24_H_14_FCl_2_N_5_S_2_, [M]+ 526.1807 Found 526.1789.

#### 3.3.12. 2-(3-(2,5-Dichloropyridin-3-yl)-1-(4-(4-fluorophenyl)thiazol-2-yl)-4,5-dihydro-1H-pyrazol-5-yl)benzo[d]thiazole (**12**)

Physical state: solid; *Rf* value: 0.64 (5:5, *n*-Hex:EtOAc); Melting Point: 226–227 °C; Colour: off-white; Yield = 49%, FT-IR (cm^−1^): 3076 (C=CHstr), 2859 (CHstr), 1663 (C=Nimine), 1618 (C=Cstr), 1445 (C=Cbend), 1247 (C-F), 1178 (C-N), 593 (C-Cl); ^1^H-NMR (600 MHz, DMSO-*d6*): δ 9.14 (s, 1H, pyridin-H), 9.02 (s, 1H, pyridin-H), 8.68 (dd, J = 8.1, 1.8 Hz, 1H, benzothiazol-H), 8.54 (dd, *J* = 7.8, 2.0 Hz, 1H, benzothiazol-H), 8.41 (d, *J* = 7.6 Hz, 2H, Ar-H), 8.28 (d, 2H, *J* = 7.3 Hz, Ar-H), 8.18 (t, *J* = 7.5 Hz, 2H, benzothiazol-H), 7.73 (s, 1H, thiazol-H), 4.87 (dd, 1H, pyrazoline-H), 3.71 (dd, 1H, pyrazoline-H), 2.76 (dd, 1H, pyrazoline-H); ^13^C-NMR (150 MHz, DMSO-*d6*): δ 172.5, 155.4, 154.2, 153.5, 151.3, 150.3, 149.4, 147.9, 145.2, 144.9, 143.9, 142.8, 139.2, 138.5, 137.7, 136.5, 135.0, 131.7, 137.8, 126.4, 124.4, 102.2, 61.6, 43.4; HREI-MS: *m/z* calcld for C_24_H_14_Cl_2_FN_5_S_2_, [M]+ 526.6538 Found 526.6507.

#### 3.3.13. 2-(1-(4-(4-(Benzyloxy)phenyl)thiazol-2-yl)-3-(2,5-dichloropyridin-3-yl)-4,5-dihydro-1H-pyrazol-5-yl)benzo[d]thiazole (**13**)

Physical state: solid; *Rf* value: 0.51 (7:3, *n*-Hex:EtOAc); Melting Point: 242–243 °C; Colour: light yellow; Yield = 58%, FT-IR (cm^−1^): 3059 (C=CHstr), 2843 (CHstr), 1671 (C=Nimine), 1628 (C=Cstr), 1439 (C=Cbend), 1263 (C-O), 1192 (C-N), 608 (C-Cl); ^1^H-NMR (600 MHz, DMSO-*d6*): δ 9.08 (s, 1H, pyridin-H), 8.82 (s, 1H, pyridin-H), 8.67 (dd, *J* = 7.9, 3.0 Hz, 1H, benzothiazol-H), 8.61 (dd, *J* = 7.7, 2.7 Hz, 1H, benzothiazol-H), 8.49 (d, *J* = 7.0 Hz, 2H, Ar-H), 8.37 (dd, *J* = 7.4, 2.0 Hz, 2H, Ar-H), 8.23 (t, *J* = 7.8 Hz, 2H, benzothiazol-H), 8.11 (t, 2H, *J* = 7.5 Hz, Ar-H), 7.86 (d, *J* = 7.2 Hz, 2H, Ar-H), 7.79 (m, 1H, Ar-H), 7.58 (s, 1H, thiazol-H), 5.43 (s, 2H, O-CH_2_), 4.76 (dd, 1H, pyrazoline-H), 3.84 (dd, 1H, pyrazoline-H), 2.81 (dd, 1H, pyrazoline-H); ^13^C-NMR (150 MHz, DMSO-*d6*): δ 177.4, 158.9, 156.4, 156.1, 153.5, 153.2, 152.4, 152.1, 150.4, 149.2, 147.6, 146.3, 146.1, 140.8, 140.2, 139.7, 138.1, 137.6, 135.8, 132.4, 131.5, 129.2, 128.0, 127.1, 126.3, 124.3, 121.9, 104.7, 72.6, 63.5, 43.1; HREI-MS: *m/z* calcld for C_31_H_21_Cl_2_N_5_OS_2_, [M]+ 614.5433 Found 614.5409.

#### 3.3.14. 2-(2-(5-(Benzo[d]thiazol-2-yl)-3-(2,5-dichloropyridin-3-yl)-4,5-dihydro-1H-pyrazol-1-yl)thiazol-4-yl)-3,5-dichlorophenol (**14**)

Physical state: solid; *Rf* value: 0.63 (5:5, *n*-Hex:EtOAc); Melting Point: 238–239 °C; Colour: pale-yellow; Yield = 64%, FT-IR (cm^−1^): 3263 (-OH), 3075 (C=CHstr), 2853 (CHstr), 1681 (C=Nimine), 1617 (C=Cstr), 1413 (C=Cbend), 1168 (C-N), 587 (C-Cl); ^1^H-NMR (600 MHz, DMSO-*d6*): δ 10.19 (s, 1H, -OH), 9.13 (s, 1H, pyridin-H), 8.94 (s, 1H, pyridin-H), 8.67 (dd, *J* = 7.5, 2.3 Hz, 1H, Benzothiazol-H), 8.51 (dd, *J* = 7.1, 1.4 Hz, 1H, Benzothiazol-H), 8.40 (t, *J* = 7.8 Hz, 2H, Benzothiazol-H), 8.19 (s, 1H, Ar-H), 8.11 (s, 1H, Ar-H), 7.52 (s, 1H, thiazol-H), 4.88 (dd, 1H, pyrazoline-H), 3.69 (dd, 1H, pyrazoline-H), 2.82 (dd, 1H, pyrazoline-H); ^13^C-NMR (150 MHz, DMSO-*d6*): δ 173.6, 154.4, 153.7, 152.5, 151.2, 150.4, 150.1, 149.2, 147.9, 145.4, 142.2, 139.2, 138.5, 136.5, 133.1, 131.2, 124.5, 122.8, 121.5, 119.1, 118.6, 104.3, 61.9, 43.3; HREI-MS: *m/z* calcld for C_24_H_13_Cl_4_N_5_OS_2_, [M]+ 593.3479 Found 593.3451.

#### 3.3.15. 2-(1-(4-(4-Bromo-3,5-dimethylphenyl)thiazol-2-yl)-3-(2,5-dichloropyridin-3-yl)-4,5-dihydro-1H-pyrazol-5-yl)benzo[d]thiazole (**15**)

Physical state: solid; *Rf* value: 0.73 (4:6, *n*-Hex:EtOAc); Melting Point: 221–222 °C; Colour: light-yellow; Yield = 45%, FT-IR (cm^−1^): 3062 (C=CHstr), 2868 (CHstr), 1683 (C=Nimine), 1623 (C=Cstr), 1432 (C=Cbend), 1151 (C-N), 594 (C-Cl); ^1^H-NMR (600 MHz, DMSO-*d6*): δ 9.07 (s, 1H, pyridin-H), 8.81 (s, 1H, pyridin-H), 8.59 (dd, *J* = 7.7, 2.0 Hz, 1H, Benzothiazol-H), 8.44 (dd, *J* = 7.2, 1.8 Hz, 1H, Benzothiazol-H), 8.32 (t, *J* = 7.9 Hz, 2H, Benzothiazol-H), 8.11 (s, 1H, Ar-H), 8.07 (s, 1H, Ar-H), 7.57 (s, 1H, thiazol-H), 4.70 (dd, 1H, pyrazoline-H), 3.74 (dd, 1H, pyrazoline-H), 2.87 (dd, 1H, pyrazoline-H), 2.41 (s, 6H, -CH_3_); ^13^C-NMR (150 MHz, DMSO-*d6*): δ 174.6, 156.9, 154.7, 152.5, 151.0, 149.9, 147.2, 146.6, 145.3, 141.5, 140.0, 138.3, 135.2, 133.9, 131.0, 130.5, 127.1, 123.6, 122.9, 116.2, 112.8, 106.8, 59.0, 43.7, 34.2, 33.7; HREI-MS: *m/z* calcld for C_26_H_18_BrCl_2_N_5_S_2_, [M]+ 615.8349 Found 615.8321.

#### 3.3.16. 2-(3-(2,5-Dichloropyridin-3-yl)-1-(4-(naphthalen-2-yl)thiazol-2-yl)-4,5-dihydro-1H-pyrazol-5-yl)benzo[d]thiazole (**16**)

Physical state: solid; *Rf* value: 0.59 (7:3, *n*-Hex:EtOAc); Melting Point: 243–244 °C; Colour: light-yellow; Yield = 54%, FT-IR (cm^−1^): 3106 (C=CHstr), 2873 (CHstr), 1672 (C=Nimine), 1634 (C=Cstr), 1413 (C=Cbend), 1164 (C-N), 588 (C-Cl); ^1^H-NMR (600 MHz, DMSO-*d6*): δ 9.03 (s, 1H, pyridin-H), 8.76 (s, 1H, pyridin-H), 8.71 (s, 1H Ar-H), 8.60 (dd, *J* = 7.2, 1.8 Hz, 1H, Ar-H), 8.43 (d, *J* = 7.2 Hz, 1H, Ar-H), 8.31 (dd, *J* = 7.2, 1.6 Hz, 1H, benzothiazol-H), 8.19 (dd, *J* = 7.6, 2.3 Hz, 1H, benzothiazol-H), 8.11 (d, *J* = 7.5 Hz, 1H, Ar-H), 7.88 (dd, *J* = 7.3, 2.5 Hz, 1H, Ar-H), 7.72 (t, *J* = 7.8 Hz, 2H, Ar-H), 7.59 (t, *J* = 7.4 Hz, 2H, benzothiazol-H), 7.63 (s, 1H, thiazol-H), 4.84 (dd, 1H, pyrazoline-H), 3.69 (dd, 1H, pyrazoline-H), 2.78 (dd, 1H, pyrazoline-H); ^13^C-NMR (150 MHz, DMSO-d6): δ 176.4, 156.0, 155.4, 154.9, 151.9, 150.4, 149.8, 148.3, 147.2, 143.3, 141.2, 141.5, 138.2, 137.0, 134.0, 133.5, 130.2, 129.4, 125.9, 121.9, 118.2, 117.3, 114.4, 111.7, 109.3, 108.4, 63.7, 44.4; HREI-MS: *m/z* calcld for C_28_H_17_Cl_2_N_5_S_2_, [M]+ 558.1962 Found 558.1943.

#### 3.3.17. 2-(1-(4-(2,3-Dichlorophenyl)thiazol-2-yl)-3-(2,5-dichloropyridin-3-yl)-4,5-dihydro-1H-pyrazol-5-yl)benzo[d]thiazole (**17**)

Physical state: solid; *Rf* value: 0.61 (4:6, *n*-Hex:EtOAc); Melting Point: 236–237 °C; Colour: light-yellow; Yield = 48%, FT-IR (cm^−1^): 3088 (C=CHstr), 2863 (CHstr), 1656 (C=Nimine), 1622 (C=Cstr), 1443 (C=Cbend), 1154 (C-N), 571 (C-Cl); ^1^H-NMR (600 MHz, DMSO-d6): δ 9.10 (s, 1H, pyridin-H), 8.83 (s, 1H, pyridin-H), 8.61 (dd, *J* = 7.4, 2.7 Hz, 1H, Benzothiazol-H), 8.54 (dd, *J* = 7.3, 1.9 Hz, 1H, Benzothiazol-H), 8.38 (dd, 1H, J = 7.9, 1.6 Hz, Ar-H), 8.21 (dd, 1H, *J* = 7.7, 1.3 Hz, Ar-H), 8.08 (t, *J* = 7.4 Hz, 2H, Benzothiazol-H), 7.83 (t, *J* = 7.7 Hz, 1H, Ar-H), 7.57 (s, 1H, thiazol-H), 4.68 (dd, 1H, pyrazoline-H), 3.73 (dd, 1H, pyrazoline-H), 2.69 (dd, 1H, pyrazoline-H); ^13^C-NMR (150 MHz, DMSO-*d6*): δ 172.3, 157.2, 154.4, 150.2, 147.2, 145.8, 144.0, 143.9, 142.4, 140.1, 139.5, 139.4, 138.6, 138.7, 137.5, 134.7, 131.5, 130.8, 127.3, 125.2, 115.9, 102.0, 63.8, 40.6; HREI-MS: *m/z* calcld for C_24_H_13_Cl_4_N_5_S_2_, [M]+ 577.2485 Found 577.2461.

### 3.4. Molecular Modelling Assay

In this study, proteins and ligands were examined using the software AutoDock 4.2.0. Models were obtained from the RCSB Protein Data Bank (PDB ID: 5NN6), which contained the 2-(2-)-2-(hydroxymethyl)piperidine-3,4,5-triol). AHA and MIG were created in the binding spaces of urease and α-glucosidase using Discovery Studio 4.0. AHAs and MIGs were then refined and hydrogens were added to the side-chains [48,49,50,51,52].

### 3.5. α-Glucosidase Inhibitory Assay

A procedure based on the previously reported procedure with minor modifications was used to determine the inhibitory capacity of the newly designed derivatives of benzothiazole-derived pyrazoline-based thiazole (**1**–**17**). For 20 min at 37 °C, 250 mL of 1.0 U/mL carbose was incubated with 500 mL of 1.0 U/mL glucose in 100 mM of phosphate buffer (pH 6.8) for 20 min. The reaction mixture was then incubated for 1 h at 37 °C with 250 mL of 4-nitrophenyl-b-D-glucopyranoside solution and 250 mL of 1% starch dissolved in 100 mM of phosphate buffer (pH 6.8), respectively. Following this, the reaction mixture was heated for 10 min with 1.0 mL of 3,5-dinitrosalicylic acid colouring reagent. The absorbance of the final reaction mixture was determined for *α*-glucosidase at 405 nm. To correct the absorbance, a blank was prepared. The positive control used was acarbose solution. A percentage of the inhibitory activity was calculated using a control sample without the inhibitors [53].

### 3.6. Urease Inhibitory Assay

In this study, newly synthesized pyrazoline-based thiazole derivatives (**1**–**17**) were evaluated for their ability to inhibit urease activity. During the experiment, 55 L of buffer solution containing 100 mM of urea was combined with 25 L of enzyme solution to prepare reaction mixtures. A volume of 5 L containing 1 mM concentrations of recently produced compounds was then added to these mixtures. After 15 min of incubation at 30 °C, the reactions were allowed to occur in 96-well plates. The detection of ammonia generation by urease was carried out by using the Indophenol technique. A phenol reagent containing 1% *w*/*v* phenol and 0.005% *w*/*v* sodium nitroprusside was added to each well along with 70 lL of an alkali reagent containing 0.5% *w*/*v* NaOH and 0.1% active chloride NaOCl. Utilizing a microplate reader manufactured by Molecular Device in the United States, the increased absorbance was measured at 630 nm after another 50 min. In triplicate, the aforementioned procedures were performed using a constant end volume of 200 mL. The final data, representing the rate at which the absorbance changed per minute, were analysed with the SoftMax Pro program by Molecular Device, New York, NY, USA. In alkaline conditions with a pH of 8.2, the experiments were conducted using 0.01 M K_2_HPO_4_·3H_2_O, 1 mM EDTA, and 0.01 M LiCl. A conventional inhibitor of urease, thiourea, was used in this study to determine the percentage of inhibition [54].

## 4. Conclusions

A series of newly benzothiazole-derived pyrazoline-based thiazole derivatives (**1**–**17**) were designed and synthesized. The synthesized benzothiazole-derived pyrazoline-based thiazole compounds were characterized through different spectroscopic techniques such as ^1^H-NMR, ^13^C-NMR, and HRMS. After that, their inhibitory activity against *α*-glucosidase and urease enzymes was evaluated. The compounds (**1**–**17**) exhibited remarkable inhibitory potential against targeted α-glucosidase and urease enzymes under the positive control of acarbose and thiourea as standard drugs, respectively. Among the synthesized series, the compounds **6**, **7**, **12,** and **14** demonstrated were found to be significantly active, as seen by their very low IC_50_ values, indicating their notable inhibitory activity. Furthermore, the structure–activity relationship (SAR) study was carried out based on various functional groups linked to the aryl group, the nature of the substituent, and positions of the substituent, which play a significant role in determining the effectiveness of these compounds as inhibitors of *α*-glucosidase and urease enzymes. In addition, to correlate the in vitro study well with the in silico study, a molecular docking study was conducted for the most active compounds and the result obtained corroborated that these active compounds established several key interactions with the active sites of targeted enzymes.

## Figures and Tables

**Table 1:** Different substituent(s) along with *α*-glucosidase and urease inhibitory activities of synthesized benzothiazole-derived pyrazoline-based thiazole (**1**–**17**).

S. No.	R	α-Glucosidase IC_50_ (µM ±SEM)	Urease IC_50_(µM ± SEM)
**1**	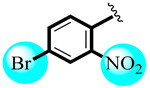	13.10 ± 0.40	41.50 ± 1.70
**2**	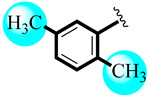	14.20 ± 0.10	37.20 ± 1.90
**3**	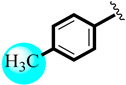	7.50 ± 0.10	33.20 ± 3.80
**4**	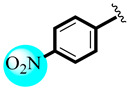	4.10 ± 0.30	23.30 ± 2.40
**5**	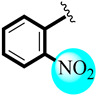	6.70 ±0.30	32.50 ± 2.10
**6**	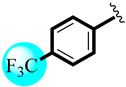	2.50 ± 0.30	14.30 ± 3.90
**7**	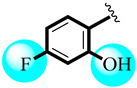	3.20 ± 0.10	19.20 ± 0.10
**8**	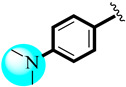	13.10 ± 0.10	39.20 ± 0.60
**9**	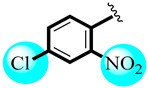	4.40 ± 0.30	29.40 ± 0.10
**10**	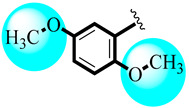	8.90 ± 0.20	33.70 ± 0.10
**11**	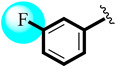	3.80 ± 0.30	25.30 ± 0.20
**12**	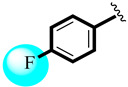	3.50 ± 0.10	22.30 ± 0.80
**13**	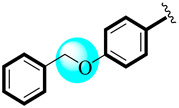	N.A *	N.A *
**14**	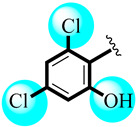	3.40 ± 0.10	21.80 ± 2.90
**15**	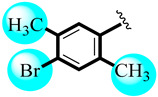	17.50 ± 0.20	38.20 ± 0.50
**16**	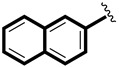	N.A *	N.A *
**17**	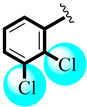	6.80 ± 0.20	31.30 ± 0.40
**Standard drug Acarbose**		5.30 ± 0.30	=
**Standard drug Thiourea**		=	31.40 ± 2.50

N.A * = Not active.

**Table 2:** Different types of interactions established by most active analogues (**6**, **7**, **12**, and **14**) of synthesized benzothiazole-derived pyrazoline-based thiazole (**1**–**17**) with their respective distance along with docking score.

Active Analogues	Receptor	Types of INTERACTIONS	Distance (A^o^)	Docking Score
Analogue-**6** in *α*-glucosidase complex	GLY-A-116	Pi-Pi T-shaped	5.16	−11.47
	TRP-A-231	Pi-Pi T-shaped	6.42	
	LEU-A-286	Pi-R	5.72	
	PHE-A-329	Pi-Pi T-shaped	6.81	
	PHE-A-329	Pi-Pi T-shaped	7.57	
	PHE-A-329	Pi-Pi T-shaped	5.53	
	ALA-A-328	Pi-R	6.14	
	ALA-A-328	Pi-R	4.61	
	TRP-A-430	Pi-R	6.08	
	GLY-A-78	H-F	5.40	
	TRP-A-82	Pi-R	6.04	
	TRP-A-82	H-B	5.16	
	HIS-A-438	H-F	5.38	
	TYR-A-332	Pi-Pi T-shaped	4.91	
	TYR-A-332	Pi-Pi T-shaped	6.35	
Analogue-**6** in Urease complex	TRP-A-84	Pi-R	5.87	
	PHE-A-330	Pi-R	5.81	
	PHE-A-330	Pi-Pi stacked	5.69	
	PHE-A-331	Pi-S	5.68	
	TYR-A-70	Pi-S	6.30	
	TRP-A-279	Pi-S	6.53	
	TRP-A-279	Pi-R	4.44	
	TRP-A-279	Pi-Pi stacked	5.20	
	ASP-A-276	H-F	6.13	
	ASN-A-280	H-F	4.97	
	ASN-A-280	H-F	4.65	
Analogue-**7** in *α*-glucosidase complex	GLY-A-116	Pi-Pi stacked	4.82	
	LEU-A-286	Pi-R	5.88	
	PHE-A-329	Pi-Pi stacked	6.92	
	THR-A-120	H-B	4.33	
	GLU-A-197	H-F	3.27	
	TYR-A-128	H-B	6.85	
Analogue-**7** in Urease complex	PHE-A-330	Pi-Pi stacked	5.62	−10.17
	PHE-A-330	Pi-R	5.82	
	TRP-A-279	Pi-Pi stacked	6.52	
	TRP-A-279	Pi-Pi stacked	5.75	
	TRP-A-279	Pi-Pi stacked	4.91	
	TYR-A-70	Pi-S	5.88	
	TYR-A-70	Pi-Pi stacked	6.72	
	TYR-A-70	Pi-Pi stacked	5.79	
	TRP-A-84	Pi-R	5.32	
	ASP-A-285	H-F	4.84	
	TYR-A-334	Pi-Pi stacked	4.73	
	PHE-A-331	Pi-S	4.45	
Analogue-**12** in *α*-glucosidase complex	PHE-A-329	Pi-S	6.72	−10.01
	PHE-A-329	Pi-Pi stacked	7.23	
	PHE-A-329	Pi-Pi stacked	6.93	
	TRP-A-331	Pi-Pi stacked	6.40	
	LEU-A-286	Pi-R	5.89	
	TRP-A-82	Pi-Pi stacked	4.62	
	TYR-A-128	H-B	6.52	
	GLU-A-197	H-F	4.57	
Analogue-**12** in Urease complex	PHE-A-330	Pi-R	3.90	−9.98
	PHE-A-330	Pi-Pi stacked	4.73	
	TYR-A-334	Pi-Sigma	4.48	
	TYR-A-334	Pi-Pi stacked	4.31	
	TYR-A-334	Pi-Pi stacked	4.30	
	TYR-A-70	Pi-S	5.60	
	ASP-A-285	H-F	4.65	
	PHE-A-331	Pi-S	4.40	
	PHE-A-331	Pi-R	5.27	
	TRP-A-279	Pi-Pi stacked	6.73	
	TRP-A-279	Pi-S	7.59	
	HIS-A-440	Pi-R	4.29	
Analogue-**14** in *α*-glucosidase complex	GLY-A-116	Pi-Pi stacked	4.90	−9.27
	TRP-A-231	Pi-Pi stacked	5.94	
	LEU-A-286	Pi-R	4.94	
	ASP-A-70	H-B	7.13	
	ASP-A-70	Pi-anion	7.47	
	MET-A-437	Pi-R	5.93	
	TYR-A-440	Pi-R	6.15	
	PHE-A-329	Pi-Pi stacked	6.89	
	PHE-A-329	Pi-anion	4.71	
	PHE-A-329	Pi-Pi stacked	5.45	
	PHE-A-329	Pi-sigma	3.49	
	TRP-A-82	Pi-R	5.43	
	ALA-A-328	Pi-R	6.05	
	TYR-A-332	Pi-Pi stacked	5.54	
	TYR-A-332	Pi-Pi stacked	6.40	
Analogue-**14** in Urease complex	PHE-A-330	Pi-S	5.65	−8.76
	PHE-A-331	Pi-R	5.66	
	TYR-A-70	Pi-Pi stacked	6.39	
	TRP-A-279	Pi-R	6.59	
	TRP-A-279	Pi-Pi stacked	4.24	
	TRP-A-279	Pi-Pi stacked	4.78	
Standard **Acarbose**	ASP-A-422	HB	4.50	
	ASP-A-630	UNFAVORABLE AA	4.74	
	ALA-A-602	HB	5.29	
	GLU-A-603	HB	4.08	
	SER-A-505	HB	3.72	
	SER-A-505	CH	3.31	
	SER-A-505	HB	3.53	
	ASN-A-496	HB	4.81	
	ILE-A-233	HB	3.71	
Standard **Thiourea**	HIS-C-315	PiS	7.23	
	THR-C-301	HB	2.73	
	THR-C-304	HB	4.28	
	GLU-C-348	HB	5.26	
	ALA-C-279	Unfavourable-DD	2.78	
	GLU-C-277	HB	3.76	
	GLY-C-280	Unfavourable-DD	3.58	
	GLY-C-280	HB	4.14	
	GLY-C-278	Unfavourable-DD	3.41	

## Data Availability

Data is contained within the article and supplementary material.

## Supplementary Materials

The following supporting information can be downloaded at: https://www.mdpi.com/article/10.3390/ph16121650/s1, Figure S1. ^1^HNMR for the compound Chalcone *(E)-3-(benzo[d]thiazol-2-yl)-1-(2,5-dichloropyridin-3-yl)prop-2-en-1-one*. Figure S2. ^13^CNMR Spectra for the compound Chalcone *(E)-3-(benzo[d]thiazol-2-yl)-1-(2,5-dichloropyridin-3-yl)prop-2-en-1-one*. Figure S3. FTIR spectra for the compound 2*(R)-2-(3-(2,5-dichloropyridin-3-yl)-1-(4-(2,5-dimethylphenyl)thiazol-2-yl)-4,5-dihydro-1H-pyrazol-5-yl)benzo[d]thiazole.* Figure S4. ^1^HNMR for the compound 2*(R)-2-(3-(2,5-dichloropyridin-3-yl)-1-(4-(2,5-dimethylphenyl)thiazol-2-yl)-4,5-dihydro-1H-pyrazol-5-syl)benzo[d]thiazole.* Figure S5. ^13^CNMR for the compound 2*(R)-2-(3-(2,5-dichloropyridin-3-yl)-1-(4-(2,5-dimethylphenyl)thiazol-2-yl)-4,5-dihydro-1H-pyrazol-5-yl)benzo[d]thiazole.* Figure S6. ^1^HNMR for the compound 3*(R)-2-(3-(2,5-dichloropyridin-3-yl)-1-(4-(p-tolyl)thiazol-2-yl)-4,5-dihydro-1H-pyrazol-5-yl)benzo[d]thiazole.* Figure S7. ^13^CNMR for the compound 3*(R)-2-(3-(2,5-dichloropyridin-3-yl)-1-(4-(p-tolyl)thiazol-2-yl)-4,5-dihydro-1H-pyrazol-5-yl)benzo[d]thiazole.* Figure S8. FTIR spectra for the compound 7*(R)-2-(2-(5-(benzo[d]thiazol-2-yl)-3-(2,5-dichloropyridin-3-yl)-4,5-dihydro-1H-pyrazol-1-yl)thiazol-4-yl)-5-fluorophenol.* Figure S9. ^1^HNMR for the compound 7*(R)-2-(2-(5-(benzo[d]thiazol-2-yl)-3-(2,5-dichloropyridin-3-yl)-4,5-dihydro-1H-pyrazol-1-yl)thiazol-4-yl)-5-fluorophenol.* Figure S10. ^13^CNMR for the compound 9*(R)-2-(1-(4-(4-chloro-2-nitrophenyl)thiazol-2-yl)-3-(2,5-dichloropyridin-3-yl)-4,5-dihydro-1H-pyrazol-5-yl)benzo[d]thiazole.* Figure S11. FTIR spectra for the compound11*(R)-2-(3-(2,5-dichloropyridin-3-yl)-1-(4-(2-fluorophenyl)thiazol-2-yl)-4,5-dihydro-1H-pyrazol-5-yl)benzo[d]thiazole.* Figure S12. FTIR spectra for the compound 14*(R)-2-(2-(5-(benzo[d]thiazol-2-yl)-3-(2,5-dichloropyridin-3-yl)-4,5-dihydro-1H-pyrazol-1-yl)thiazol-4-yl)-3,5-dichlorophenol.*
