# Supplementary material for: The Synthesis, In Vitro Bio-Evaluation, and In Silico Molecular Docking Studies of Pyrazoline–Thiazole Hybrid Analogues as Promising Anti-α-Glucosidase and Anti-Urease Agents
Source: Pharmaceuticals (Basel). 2023 Nov 25;16(12):1650. doi: 10.3390/ph16121650 (PMC10747725; doi:10.3390/ph16121650)
Supplement: Supplementary file 1 [file pharmaceuticals-16-01650-s001.zip › pharmaceuticals-2702094-supplementary.pdf]

pharmaceuticals

MDPI

Article

The Synthesis, In Vitro Bio-Evaluation, and In Silico Molecular Docking Studies of Pyrazoline–Thiazole Hybrid Analogues as Promising Anti-α-Glucosidase and Anti-Urease Agents

Yousaf Khan [1], Shoaib Khan [2], Rafaqat Hussain [3,*], Aneela Maalik [1,*], Wajid Rehman [3], Mohamed W. Attwa [4], Rafia Masood [1], HanyW. Darwish [4] and Hazem A. Ghabbour [5]

[1] Department of Chemistry, COMSATS University Islamabad Campus, Islamabad 45550, Pakistan; yousaf7n@gmail.com (Y.K.); rafia12@yahoo.com (R.M.)
[2] Department of Chemistry, Abbottabad University of Science and Technology (AUST), Abbottabad 22500, Pakistan; shoaibkhanswati@gmail.com
[3] Department of Chemistry, Hazara University, Mansehra 21120, Pakistan; sonowaj@yahoo.com
[4] Department of Pharmaceutical Chemistry, College of Pharmacy, King Saud University, Riyadh 11451, Saudi Arabia; mzeidan@ksu.edu.sa (M.W.A.); hdarwish@ksu.edu.sa (H.W.D.)
[5] School of Health and Biomedical Sciences, RMIT University, Melbourne 3083, Australia; hazem.ghabbour@rmit.edu.au
***** Correspondence: rafaqathussain0347@gmail.com (R.H.); aneela.maalik@comsats.edu.pk (A.M.)

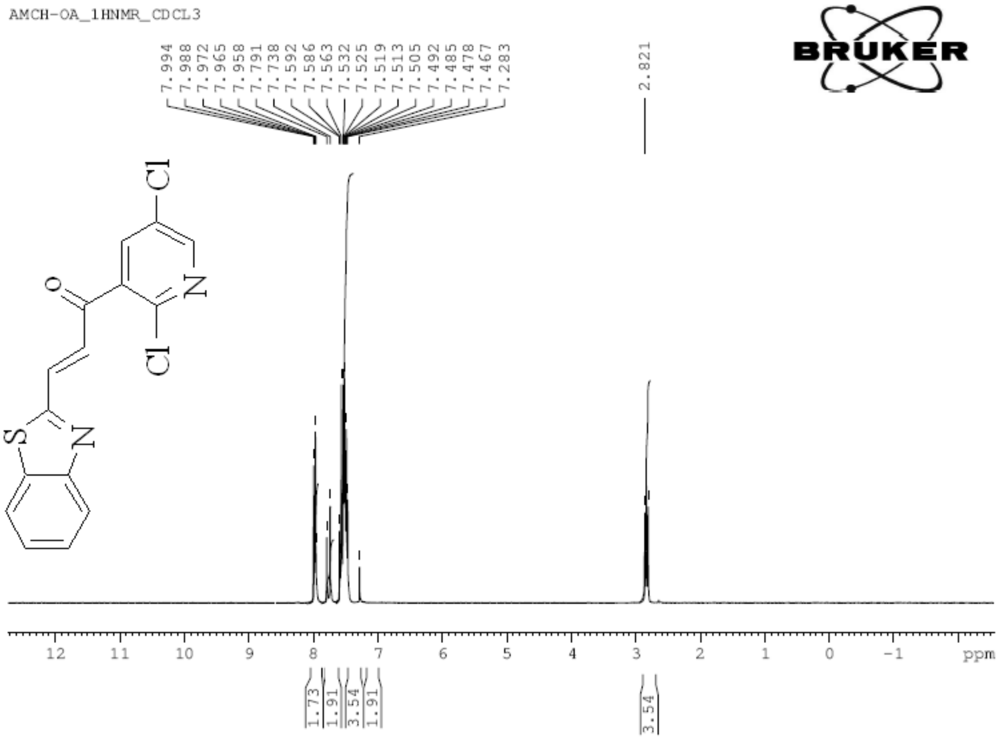

Figure S1. ^{1}HNMR for the compound **Chalcone** *(E)-3-(benzo[d]thiazol-2-yl)-1-(2,5-dichloropyridin-3-yl)prop-2-en-1-one*

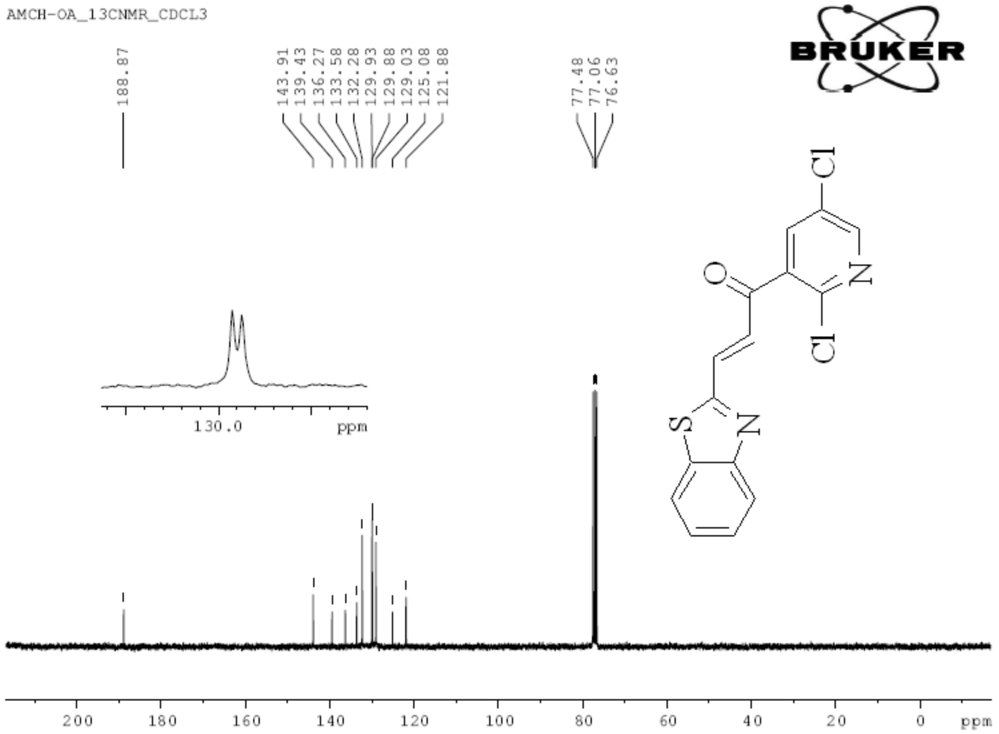

Figure S2.^{13}CNMR Spectra for the compound **Chalcone** *(E)-3-(benzo[d]thiazol-2-yl)-1-(2,5-dichloropyridin-3-yl)prop-2-en-1-one*

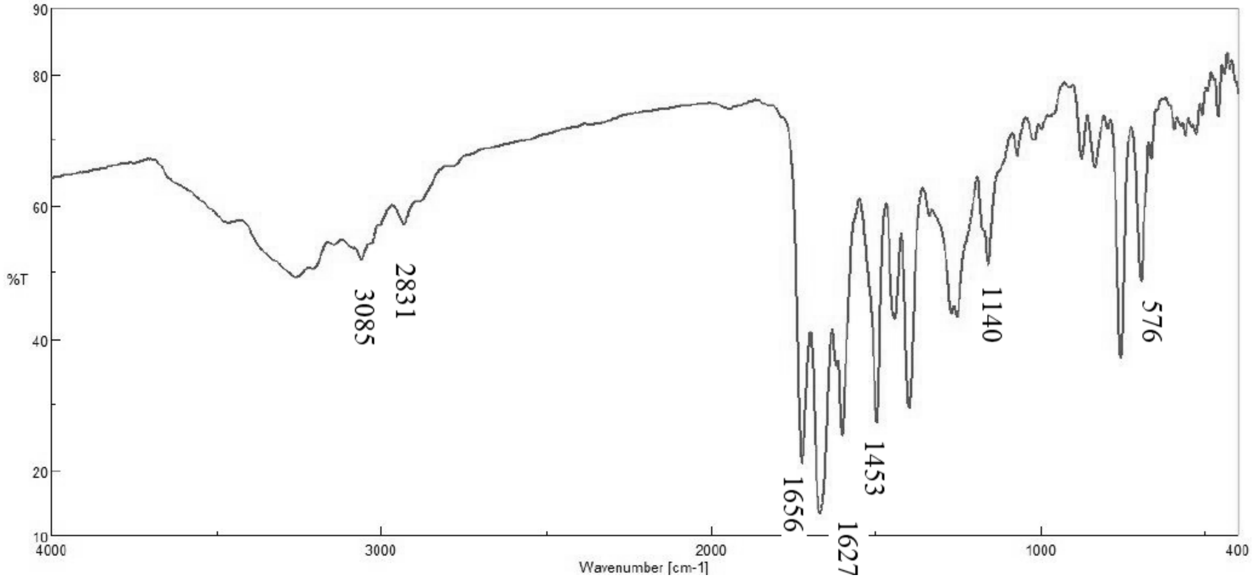

Figure S3.FTIR spectra for the compound **2***(R)-2-(3-(2,5-dichloropyridin-3-yl)-1-(4-(2,5-dimethylphenyl)thiazol-2-yl)-4,5-dihydro-1H-pyrazol-5-yl)benzo[d]thiazole*

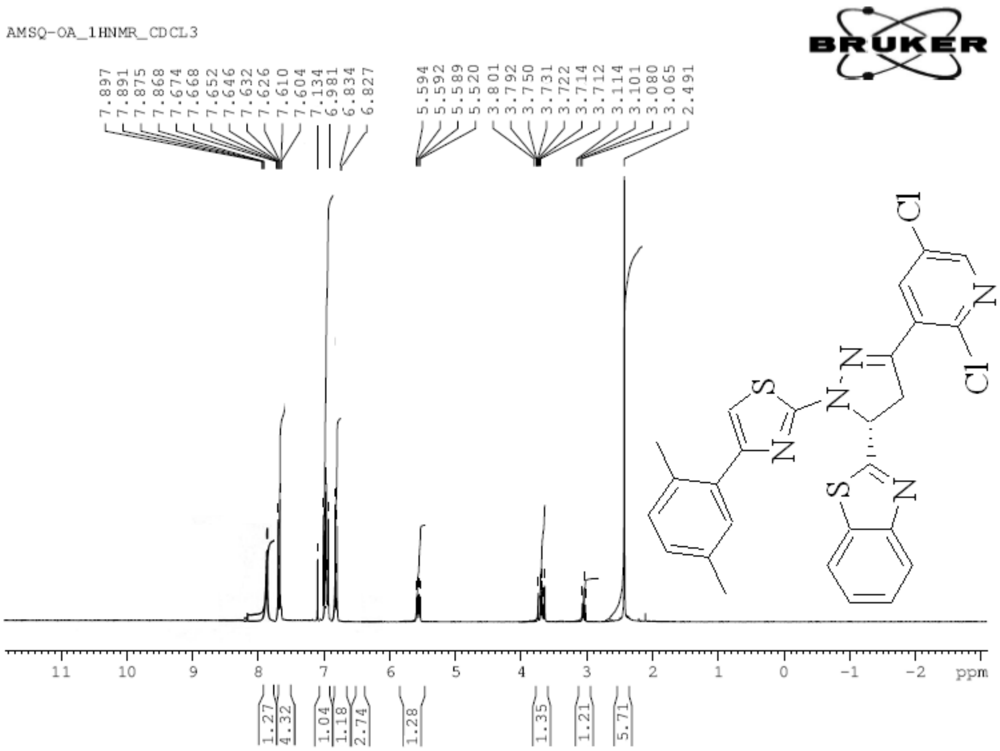

Figure S4.^{1}HNMR for the compound **2***(R)-2-(3-(2,5-dichloropyridin-3-yl)-1-(4-(2,5-dimethylphenyl)thiazol-2-yl)-4,5-dihydro-1H-pyrazol-5-yl)benzo[d]thiazole*

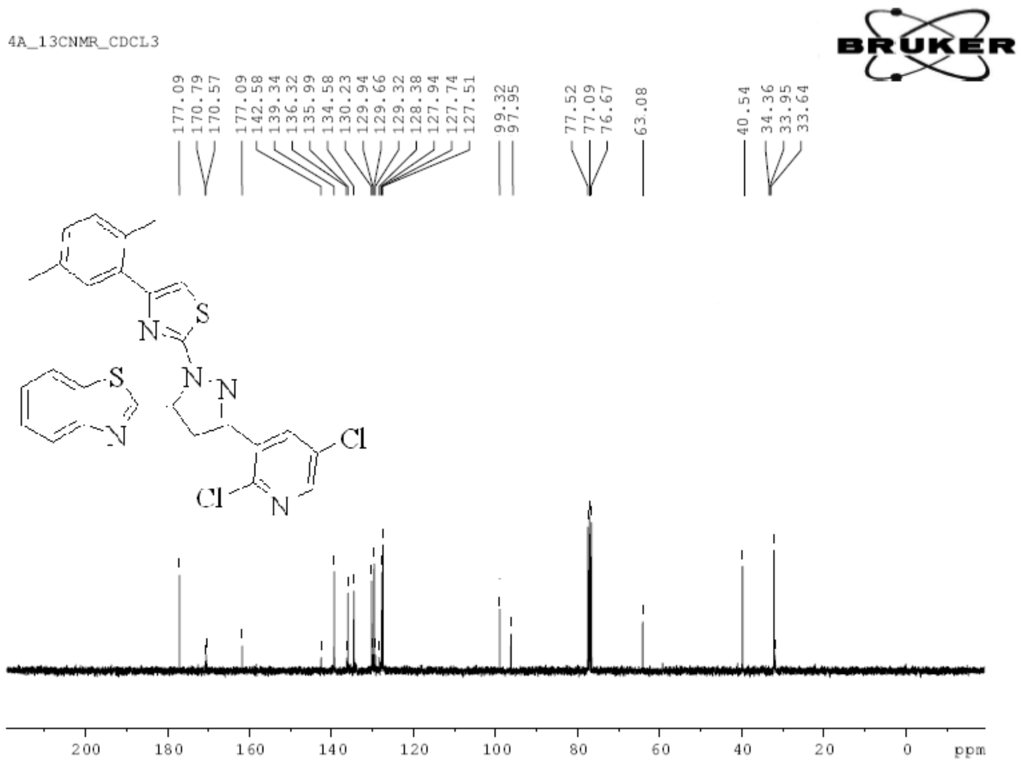

Figure S5.^{13}CNMR for the compound **2**(*R)-2-(3-(2,5-dichloropyridin-3-yl)-1-(4-(2,5-dimethylphenyl)thiazol-2-yl)-4,5-dihydro-1H-pyrazol-5-yl)benzo[d]thiazole*

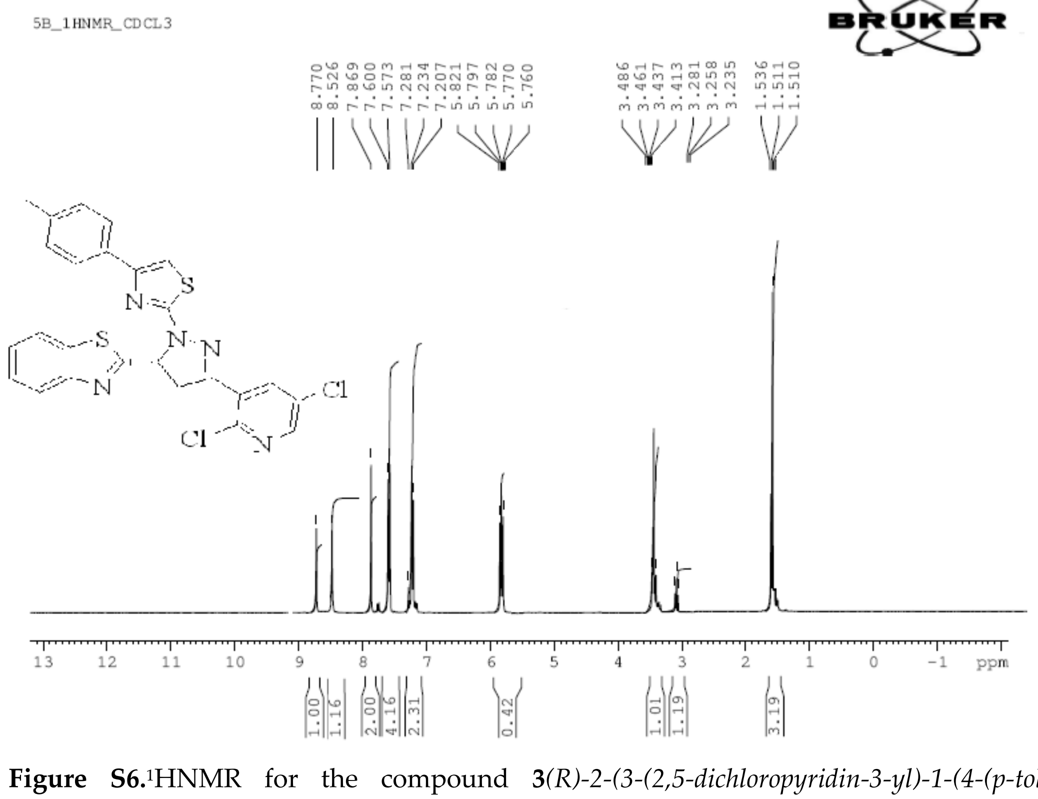

Figure S6.^{1}HNMR for the compound **3**(*R)-2-(3-(2,5-dichloropyridin-3-yl)-1-(4-(p-tolyl)thiazol-2-yl)-4,5-dihydro-1H-pyrazol-5-yl)benzo[d]thiazole*

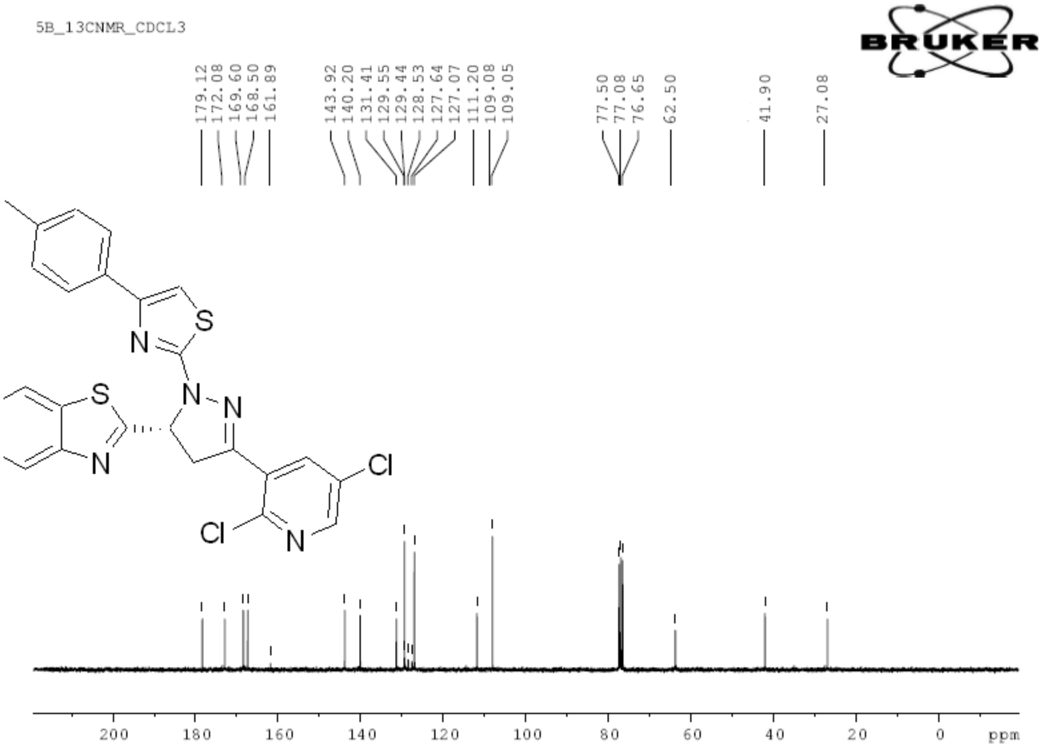

Figure S7.^{13}CNMR for the compound **3***(R)-2-(3-(2,5-dichloropyridin-3-yl)-1-(4-(p-tolyl)thiazol-2-yl)-4,5-dihydro-1H-pyrazol-5-yl)benzo[d]thiazole*

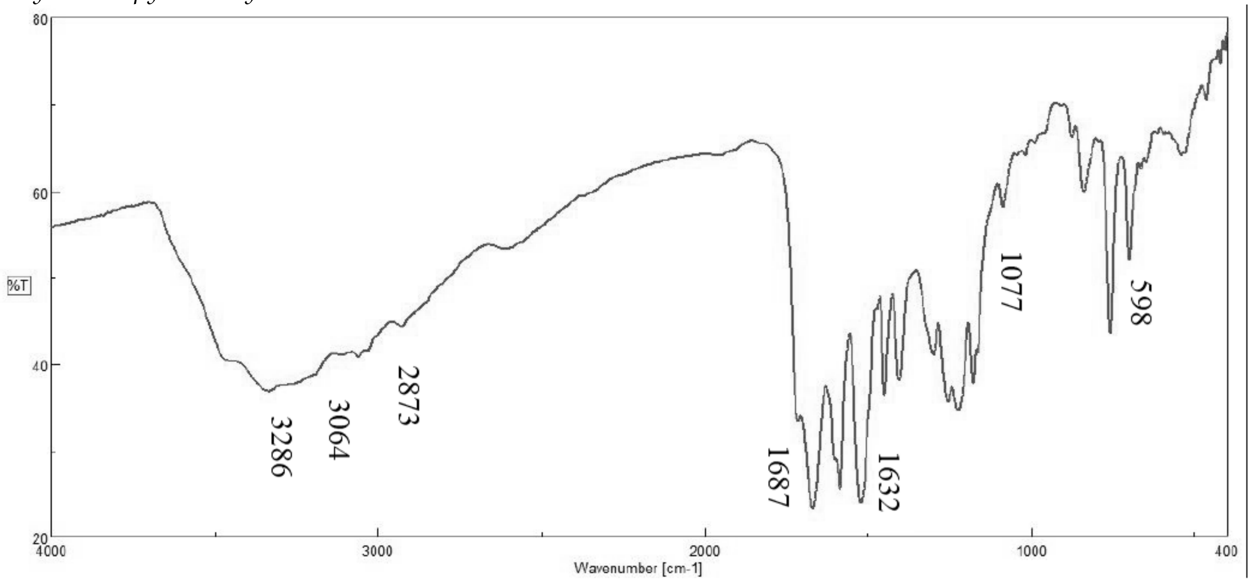

Figure S8, FTIR spectra for the compound **7***(R)-2-(2-(5-(benzo[d]thiazol-2-yl)-3-(2,5-dichloropyridin-3-yl)-4,5-dihydro-1H-pyrazol-1-yl)thiazol-4-yl)-5-fluorophenol*

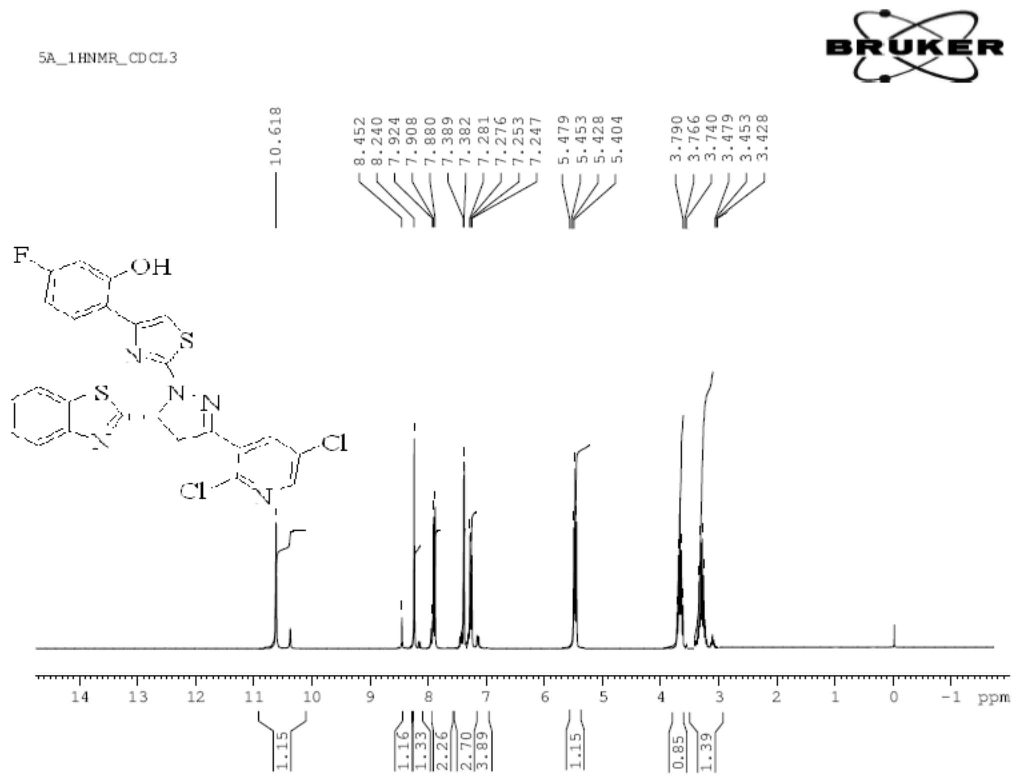

Figure S9.^{1}HNMR for the compound **7***(R)-2-(2-(5-(benzo[d]thiazol-2-yl)-3-(2,5-dichloropyridin-3-yl)-4,5-dihydro-1H-pyrazol-1-yl)thiazol-4-yl)-5-fluorophenol*

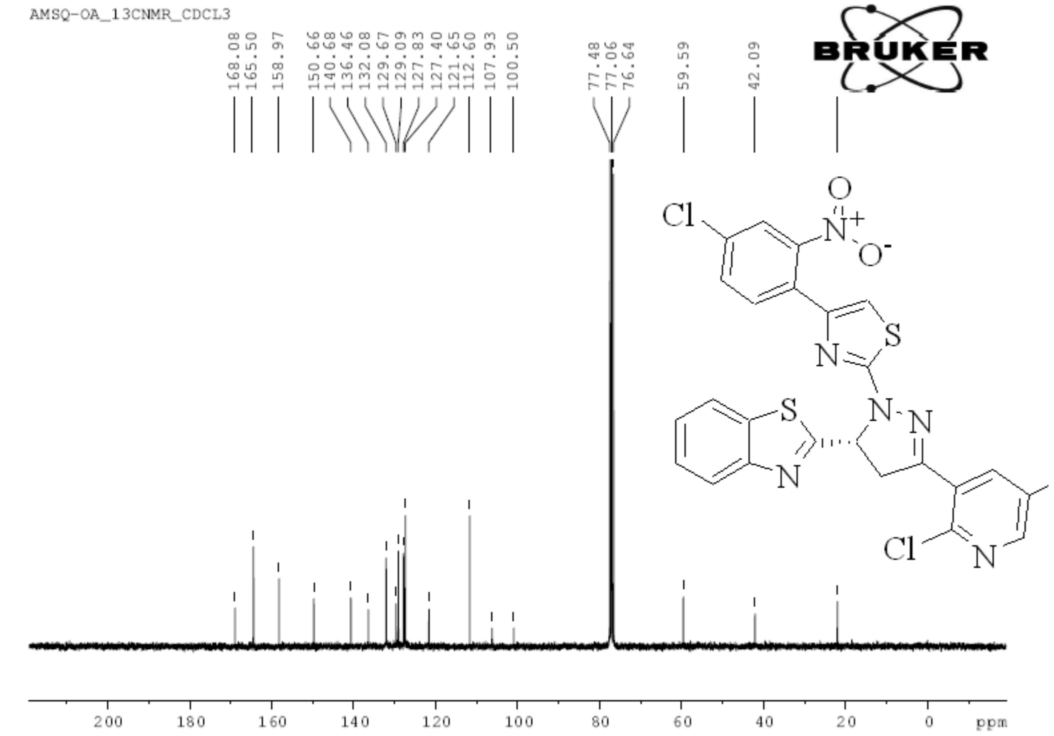

Figure S10.^{13}CNMR for the compound **9***(R)-2-(1-(4-(4-chloro-2-nitrophenyl)thiazol-2-yl)-3-(2,5-dichloropyridin-3-yl)-4,5-dihydro-1H-pyrazol-5-yl)benzo[d]thiazole*

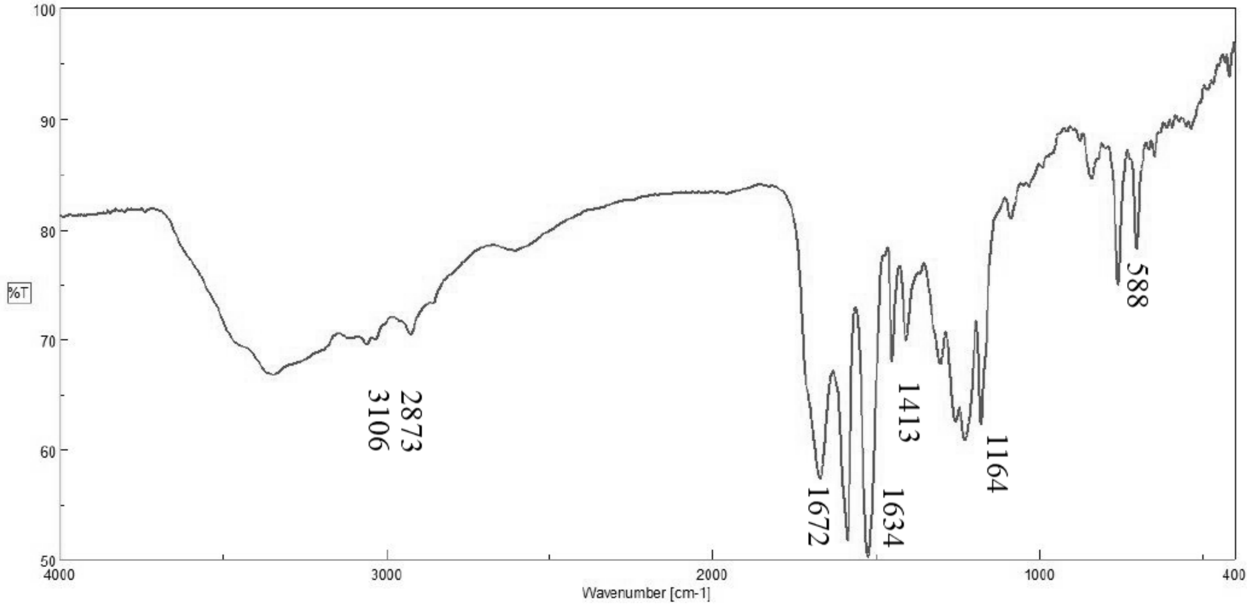

Figure S11.FTIR spectra for the compound**11***(R)-2-(3-(2,5-dichloropyridin-3-yl)-1-(4-(2-fluorophenyl)thiazol-2-yl)-4,5-dihydro-1H-pyrazol-5-yl)benzo[d]thiazole*

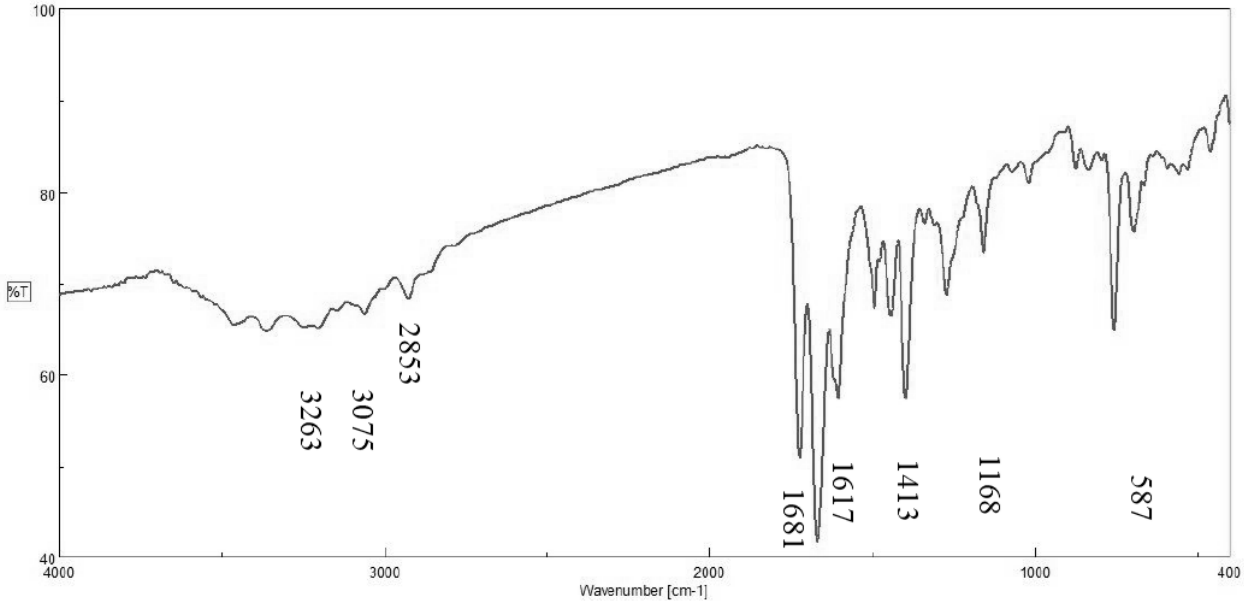

Figure S12.FTIR spectra for the compound **14***(R)-2-(2-(5-(benzo[d]thiazol-2-yl)-3-(2,5-dichloropyridin-3-yl)-4,5-dihydro-1H-pyrazol-1-yl)thiazol-4-yl)-3,5-dichlorophenol*
